# Anthropogenic disturbance keeps the coastal seafloor biogeochemistry in a transient state

**DOI:** 10.1038/s41598-018-23925-y

**Published:** 2018-04-03

**Authors:** Sebastiaan van de Velde, Vera Van Lancker, Silvia Hidalgo-Martinez, William M. Berelson, Filip J. R. Meysman

**Affiliations:** 1 Analytical, Environmental and Geo-Chemistry, Department of Chemistry, Vrije Universiteit Brussel, 1050 Brussel, Belgium; 2 Ecosystem Management Research Group, Department of Biology, Universiteit Antwerpen, 2160 Antwerpen, Belgium; 3 Royal Belgian Institute of Natural Sciences. Operational Directorate Natural Environment, 1200 Brussel, Belgium; 4 Earth Science Department, University of Southern California, CA 90089-0740 Los Angeles, USA

## Abstract

Coastal sediments and continental shelves play a crucial role in global biogeochemistry, as they form the prime site of organic carbon burial. Bottom trawling and dredging are known to increasingly impact the coastal seafloor through relocation and homogenisation of sediments, yet little is known about the effects of such anthropogenic sediment reworking on the overall cycling of carbon and other elements within the coastal seafloor. Here, we document the transient recovery of the seafloor biogeochemistry after an *in situ* disturbance. Evidence from pore-water data and model simulations reveal a short-term increase in the overall carbon mineralisation rate, as well as a longer-term shift in the redox pathways of organic matter mineralisation, favouring organoclastic sulphate reduction over methane formation. This data suggests that anthropogenic sediment reworking could have a sizeable impact on the carbon cycle in cohesive sediments on continental shelves. This imprint will increase in the near future, along with the growing economic exploitation of the coastal ocean.

## Introduction

The human imprint on the seafloor has rapidly increased in recent times^1^. Bottom trawl fishing intensity has seen a rapid global expansion since the 1950s in order to meet an increasing global food demand^2^, and although the highest trawling intensities are found in shallow coastal waters, the depletion of traditional fish stocks combined with technical advancements make that bottom trawling is expanding into deeper waters^3^. Equally, dredging activities within the coastal zone have greatly intensified in recent decades in connection to harbour extension works, maintenance and deepening of navigable waterways, land reclamation, coastal protection, and energy provision through the construction of wind farms^4^. Globally, dredging involves the excavation of >2 billion tons of sediment per year, of which ~80% is redeposited in the marine environment^5^. Up until now, research efforts have been primarily focused on the effects of bottom trawling and dredging on marine biodiversity and ecology^6–9^, as well as the effects of sediment deposition on trace metal availability^10–13^ and nutrient release^14,15^. Some work has been performed on disturbances of the slow accumulating abyssal sediments within the context of deep-sea mining^16^. Yet little is known about the direct impact of bottom trawling and dredging on the cycling of carbon and other major elements within the coastal seafloor, which is a major knowledge gap, as coastal sediments and continental shelves play a crucial role in the global carbon cycle^17–19^.

Coastal waters have a shallow water column and support high primary production, which implies that underlying sediments receive high amounts of fresh, reactive organic matter. This translates into high rates of organic matter mineralisation^20^ and as a result, thermodynamically favourable electron acceptors (oxygen, nitrate, metal oxides) are rapidly depleted within the first few millimetres to centimetres^20,21^. This makes organoclastic sulphate reduction and methanogenesis the most important mineralisation pathways in coastal sediments, leading to a large production of reduced sulphide^22^ and methane^23^. At the same time, the rapid depletion of oxygen also favours the preservation and burial of organic carbon in coastal systems. Combined with an input of refractory organic matter from the nearby land, this makes coastal sediments an essential sink for organic carbon^18,24^. The lack of data regarding the effects of bottom trawling and dredging on coastal seafloor biogeochemistry directly translates into uncertainties in global carbon budgets and hampers our understanding of the anthropogenic influence on the carbon cycle^17,18^.

Marine sediments are homogenised by bioturbation, where sediment mixing results from the burrowing, locomotion and feeding activities of sedimentary infauna^25^, as well as physical processes, such as erosion/resuspension events, which are driven by currents and waves. These natural forms of sediment homogenisation are known to have a critical impact on the cycling and burial of carbon^26,27^, and it has been proposed that changes in the biological mixing of marine sediments may even have influenced atmospheric levels of CO_2_ and O_2_ over geological time scales^28^. Given this, the question is to what extent anthropogenic sediment reworking, induced by activities like bottom trawling and dredging, has an impact on the present-day coastal carbon cycle.

Here, we provide field data that were collected before and after an anthropogenically influenced seafloor homogenisation event. This dataset was subsequently analysed by reactive-transport modelling. Our results reveal that anthropogenic disturbance of the seafloor strongly alters the respiration pathways of organic carbon mineralisation, and show the large time scale variation over which these pathways recover.

## Materials and Methods

### Sediment sampling

Sediment cores were collected with a single core gravity corer (UWITEC, Austria) using transparent PVC core liners (inner diameter: 6 cm; length: 60 cm). Sediment cores were inspected for disturbances, and only cores with an apparent undisturbed sediment-water interface were kept for analysis. Intact sediment cores (n = 12) were transported back to the shore-based laboratory in a thermally insulated container (transit time ~ 2 hr).

Upon arrival at the laboratory, the cores were submerged in an air-saturated water tank in a climate-controlled room at *in situ* temperature, filled with bottom water from the sampling site and left overnight. The next day, 2 cores were randomly selected and sectioned for pore water collection in a nitrogen filled glove box (Coy lab products, USA). Sediment cores were sectioned at 0.5 cm resolution for the first couple of centimetres, and then 1-2 cm resolution till the end of the core.

Sediment slices were collected in 50 ml centrifuge tubes (polypropylene; TPP, Switzerland) and centrifuged at 4000 g for 7.5 min (Sigma 3-18KS, Sigma Laborzentrifugen GmbH, Germany). Subsequently, the centrifuge tubes were opened in the anaerobic glove box and overlying pore water was transferred into suitable sample containers after filtration through 0.4 µm cellulose filters (Chromafil Xtra). Pore-water samples were analysed for the DIC, nutrients (NH_4_^+^, PO_4_^3−^), dissolved Fe, dissolved Mn and sulphate (SO_4_^2−^). The solid phase that remained after centrifugation was freeze-dried and manually ground with a pestle and mortar for solid phase analysis (see Supplementary Information).

### Pore water analysis

Samples for major cation analysis (dissolved Fe, dissolved Mn) were fixed with 50 µL per mL of sub-boiled distilled HNO_3_ (65%) and preserved at 4 °C. Prior to analysis, these preserved samples were diluted 50 times with a standard matrix solution containing 35% artificial seawater, 2% HNO_3_ and 0.2 mg L^−1^ Ytterbium as an internal standard^29^. Samples were analysed by Inductively Coupled Plasma – Optical Emission Spectroscopy (ICP-OES, ThermoFisher iCAP6500).

Sulphate (SO_4_^2−^) samples were diluted 10 times prior to analysis. Separation was done by ion chromatography, using an isocratic eluent (3.5 mM Na_2_CO_3_/1 mM NaHCO_3_) combined with Dionex AS-14 analytical column (Thermo Scientific). Detection was done by a conductivity detector (ED40 electrochemical detector)^30^.

Nutrient samples (NH_4_^+^, PO_4_^3−^) were fixed with 100 µL per mL H_2_SO_4_ (1 M) to prevent the oxidation and flocculation of reduced iron compounds, diluted 25 times with a low nutrient seawater matrix solution, and analysed by a SEAL QuAAtro segmented flow analyzer^31^.

Pore-water DIC analysis was performed using a Segmented Flow Analyser (San +  + SKALAR)^32^. Quality assurance involved regular analysis of Certified Reference Materials (CRM) which was obtained from the Scripps Institution of Oceanography (batch 140)^33^.

## Results and Discussion

The shallow Southern North Sea forms a prime example of a coastal system with an expanding anthropogenic imprint. Sediments are frequently affected by bottom trawling (1-3 times on average per year)^3^, and substantial dredging activities take place, in connection with aggregate extraction (e.g., for large beach nourishment programs), and maintenance of the major European seaports of Rotterdam, Antwerp and Hamburg^4^. The dredged sediments are disposed at dedicated locations in the coastal zone^4^.

We investigated the sediment biogeochemistry at a field site in the shallow Southern North Sea, ~5 km offshore the Belgian coast (station BCZ130, N 51°16.3′, E 2°54.3′), and nearby such a sediment disposal site (<2 km). During 7 consecutive campaigns throughout 2014 the upper sediment column was sampled. A major sediment disturbance was observed between the May and June campaigns, which strongly affected the upper ~15 cm of the sediment and heavily impacted the geochemical cycling. The cause of the disturbance was not immediately clear, although bottom trawling and dredging-related sediment disposal were prime candidates. To elucidate the possible cause and consequences, we collected pore water and solid phase data before and after the disturbance event, tracking the changes in the sediment biogeochemistry, and analysed the resulting dataset with a reactive transport model.

### Geochemical state prior to disturbance

**Figure 1:**
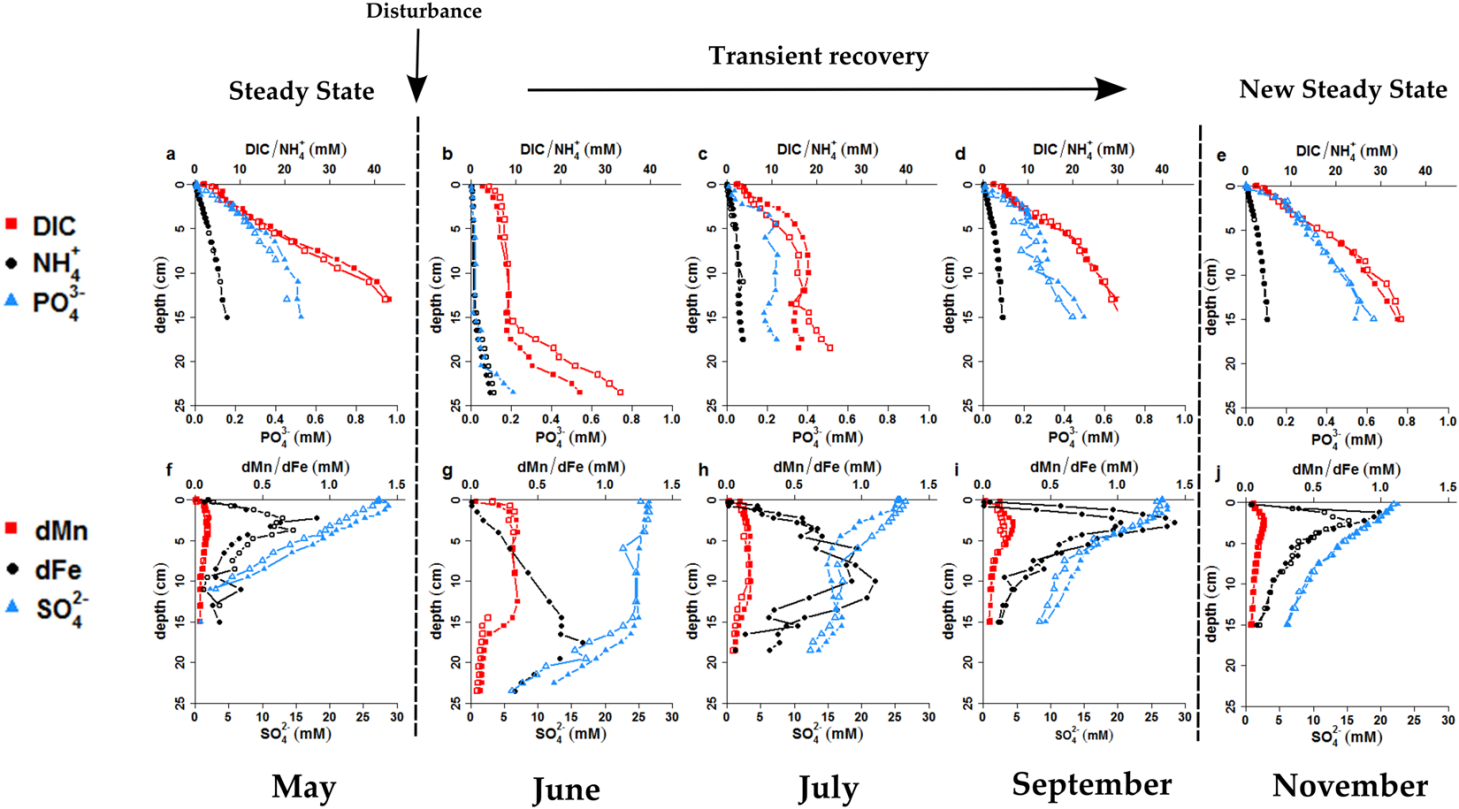
Pore-water profiles of (**a**,**b**,**c**,**b**) Dissolved Inorganic Carbon (DIC), ammonium (NH_4_^+^) and phosphate (PO_4_^3−^) and (**e**,**f**,**g**,**h**) dissolved iron (dFe), dissolved manganese (dMn) and sulphate (SO_4_^2−^) for May, June, July, September and November 2014.

Sediments near station BCZ130 are generally composed of fine-grained mud, rich in organic matter and carbonate^34^, and with little bioturbation^35^. In the 5 months prior to the disturbance, the pore-water data showed highly similar depth profiles (Fig. 1a,f and SI 1), suggesting that the sediment biogeochemistry was in or near steady state. A sediment biogeochemical model (see SI 1.2 for details) provided an excellent fit to the pore-water data, and generated estimates of biogeochemical fluxes and rates. The depth profiles of mineralisation end-products (dissolved inorganic carbon, ammonium and phosphate) attained a concave shape with depth, reflecting the combined effect of production due to organic matter mineralisation (OMM) and upward diffusion (Fig. 1a). Strong sulphate consumption (7.8 mmol S m^−2^ d^−1^) led to a shallow sulphate-methane transition zone at ~11 cm depth (Fig. 1f). The correlation between pore-water SO_4_^2−^ and DIC (elemental ratio C:S = 1.3; Fig. S2c) indicated that sulphate was consumed by both organoclastic sulphate reduction (C:S = 2), as well as by the anaerobic oxidation of methane diffusing upward (C:S = 1). The total mineralisation rate of organic matter was estimated at 16.4 mmol C m^−2^ d^−1^ of which only 0.6% occurred via aerobic respiration, 47.5% via organoclastic sulphate reduction and 51.9% was attributed to methanogenesis. The dominant oxidation pathway of free sulphide in May was electrogenic sulphur oxidation (e-SOx), whereby long filamentous cable bacteria use long-distance electron transport to couple sulphide oxidation in deeper sediment layers to oxygen reduction near the sediment-water interface^36^. Cable bacteria were detected in the top 2–7 cm of the sediment^34^, and the strong subsurface maximum of dissolved iron (dFe) and manganese (dMn) in the upper 5 cm (Fig. 1f). These depth profiles are consistent with the geochemical fingerprint of e-SOx (see refs^34,37^ for a more detailed discussion of impact of this microbial metabolism on the geochemistry at the field site).

### Disturbance and transient recovery

Between the monthly sampling visits of May and June, a major disturbance event occurred, which was directly noted upon core inspection. Sediment cores retrieved during the five previous months all showed a marked colour transition within the first 2–3 millimetres, from yellow-brown to cohesive grey clay, tightly linked to the oxygen penetration depth (Fig. S3a and ref.^34^). Yet, in the sediment cores retrieved in June, the sticky grey clay started only below ~15 cm depth, and on top, a distinct layer of less cohesive light-brown sediment was present (Fig. S3b, see Supplementary Information for a detailed description of the sediment before and after the disturbance). The pore-water data suggested a complete ‘reset’ of the pore-water geochemistry within the upper 15 cm. Depth profiles of dissolved inorganic carbon (DIC; 6.5 ± 1.8 mM), ammonium (NH_4_^+^; 0.5 ± 0.3 mM), phosphate (PO_4_^3−^; 13 ± 8 µM) and sulphate (SO_4_^2−^; 25 ± 1 mM) showed near-constant values within the upper 15 cm, with concentrations approaching those of the overlying water column (Fig. 1b,g). Dissolved manganese also showed a constant concentration (0.27 ± 0.02 mM) with depth, but was clearly enriched with respect to the overlying water. The maximum concentration of dFe appeared just below 15 cm (0.83 mM at 17.5 cm depth), resembling the subsurface peak in dFe that was present below the sediment-water interface before the disturbance (Fig. 1g).

Over the three month period after the disturbance (July-September), the DIC, NH_4_^+^ and PO_4_^3−^ depth profiles gradually recovered, driven by the mineralisation of organic matter, until they attained the concave shape and the same pore-water inventory as seen before the disturbance event (Fig. 1d and SI 4, Table S1). Interestingly, the strong concave down shape (‘bump’ in the profile) of the DIC and NH_4_^+^ profiles indicates a higher mineralisation rate in the upper 15 cm, as compared to the lower sediment (Fig. 1c,d). The ratio between DIC and NH_4_^+^ follows the Redfield stoichiometry (C:N = 106:16) before the disturbance (May), as well as during the transient recovery (July to September) (Fig. S2a). In contrast, the DIC to phosphate ratio remained well below the Redfield ratio in May and July-September (C:P = 54–72), suggesting preferential phosphate release. This was not the case in June (C:P = 101), when phosphate was apparently retained in the sediment (Fig. S2b).

The pore-water profiles of dissolved manganese, dissolved iron and sulphate suggest that during the transient recovery, manganese oxide reduction (MR), dissimilatory iron reduction (DIR) and organoclastic sulphate reduction (SR) were consecutively the dominant mineralisation pathway. As such, organic matter mineralisation followed the classical redox cascade based on thermodynamic energy gain^38^. Moreover, the observed release of dissolved manganese and iron to the pore water suggests a substantial input of oxidised manganese and iron minerals during the perturbation event. The strong enrichment of dMn in the pore water (0.27 ± 0.02 mM) in June (Fig. 1g) is consistent with the reduction of manganese (hydr)oxides, while the elevated C:P ratio in June is most likely caused by adsorption and retention of phosphate onto newly supplied iron (oxyhydr)oxides^39^. The linear increase of dFe within the upper 15 cm suggests that dissimilatory iron reduction is inhibited by MnO_2_ in the upper 15 cm^38^ and ferrous iron is supplied by diffusional transport from below. In July, dMn values decreased as likely the stock of newly introduced MnO_2_ became depleted. Mn^2+^ was removed from the pore water, most likely by co-precipitation with carbonates, which acts as a sink of reduced manganese in North-Sea sediments^40^. At the same time, dissolved iron concentrations increased (maximum value ~1 mM at 10 cm depth), stimulated by the reduction of iron oxy(hydr)oxides. The synchronous decrease of the sulphate concentration in the same zone suggests that iron oxide reduction and organoclastic sulphate reduction occurred simultaneously (Fig. 1h). In September, the depth profiles of both manganese and iron showed a prominent subsurface maximum, while sulphate showed production in the upper few centimetres (Fig. 1i). These features suggest the redevelopment of a cable bacteria population and the re-establishment of e-SOx^41^ (Fig. S5).

Pore-water profiles of DIC, NH_4_^+^, PO_4_^3−^, SO_4_^2−^, dFe and dMn did not further change between September and November (Fig. 1d,e and i,j), suggesting that the system attained a new steady state. Important to note is that all solute profiles in November were nearly identical to the profiles in May, before the disturbance event, apart from SO_4_^2−^. In May SO_4_^2−^ was depleted at ~11 cm depth (Fig. 1f), while in November, the SO_4_^2−^ concentration at 15 cm depth was still ~5 mM (Fig. 1j). Accordingly, the sulphate-methane transition zone was considerably deeper than before the disturbance (Fig. 1j), indicating the sulphur cycle was still transiently recovering.

### The cause of the disturbance

There are three possible processes acting in the Belgian coastal zone^3,42–44^ that individually, or in combination, can cause a disturbance of the seafloor as observed here; (i) a natural mud accumulation event, (ii) bottom trawling, and (iii) sediment deposition linked to dredging.

The tidal regime in the Belgian coastal zone is semi-diurnal with a mean tidal range of 4.3 m and 2.8 m at spring and neap tide, respectively. Strong rectilinear currents (>1 m s^−1^) prevail, inducing an important tidal mixing. Mud is naturally occurring in the area, with thin fluffy layers mostly being observed under neap tidal conditions and slack tide^42^. Fluffy layers of a few cm thick (~1–3 cm) are only observed occasionally, e.g., after a storm period^44^. The thickness of our disturbed sediment layer (>15 cm) largely exceeds these sediment accretion rates from natural processes, suggesting an anthropogenic origin. The Southern North Sea has a high intensity of trawling with a disturbance frequency of >1 per year on average^3^. Closer to shore, trawling intensities are higher, and for the study site, a trawling frequency of nearly 3 per year was estimated for 2014^3^. Depending on the design of the trawl and the softness of the sediment trawled, up to 35 cm of sediment can be affected by a passing trawl^45^. This range hence encompasses the observed extent of the disturbance (15 cm), and although bottom trawling is officially not allowed at the field site, the observed sediment disturbance could be caused by bottom trawling. Alternatively, the field site is located nearby (<2 km) an official disposal ground, where very soft sediments originating from dredging activities are dumped (Fig. S6; the field site lies on a straight line between the harbour and the disposal ground). A fluid mud layer can be formed when dredged material is deposited onto the seafloor^43^, and under the action of strong currents and waves, such a fluid mud layer can be transported away from the dredge location and deposited elsewhere. Analysing hydro-meteorological conditions throughout the present sampling period showed that we sampled after a stormy period, with a prolonged period of higher waves (>1 m) consistently originating from the north (NNW-NNE) (Fig. S7). Sampling also took place under neap tidal conditions, when the chance of a deposition event is highest (Fig. S7). Still, there was no official record of any sediment disposal by dredging contractors in the weeks prior to the observed disturbance (Fig. S7), and so the actual cause of the disturbance could not be pinpointed.

**Figure 2:**
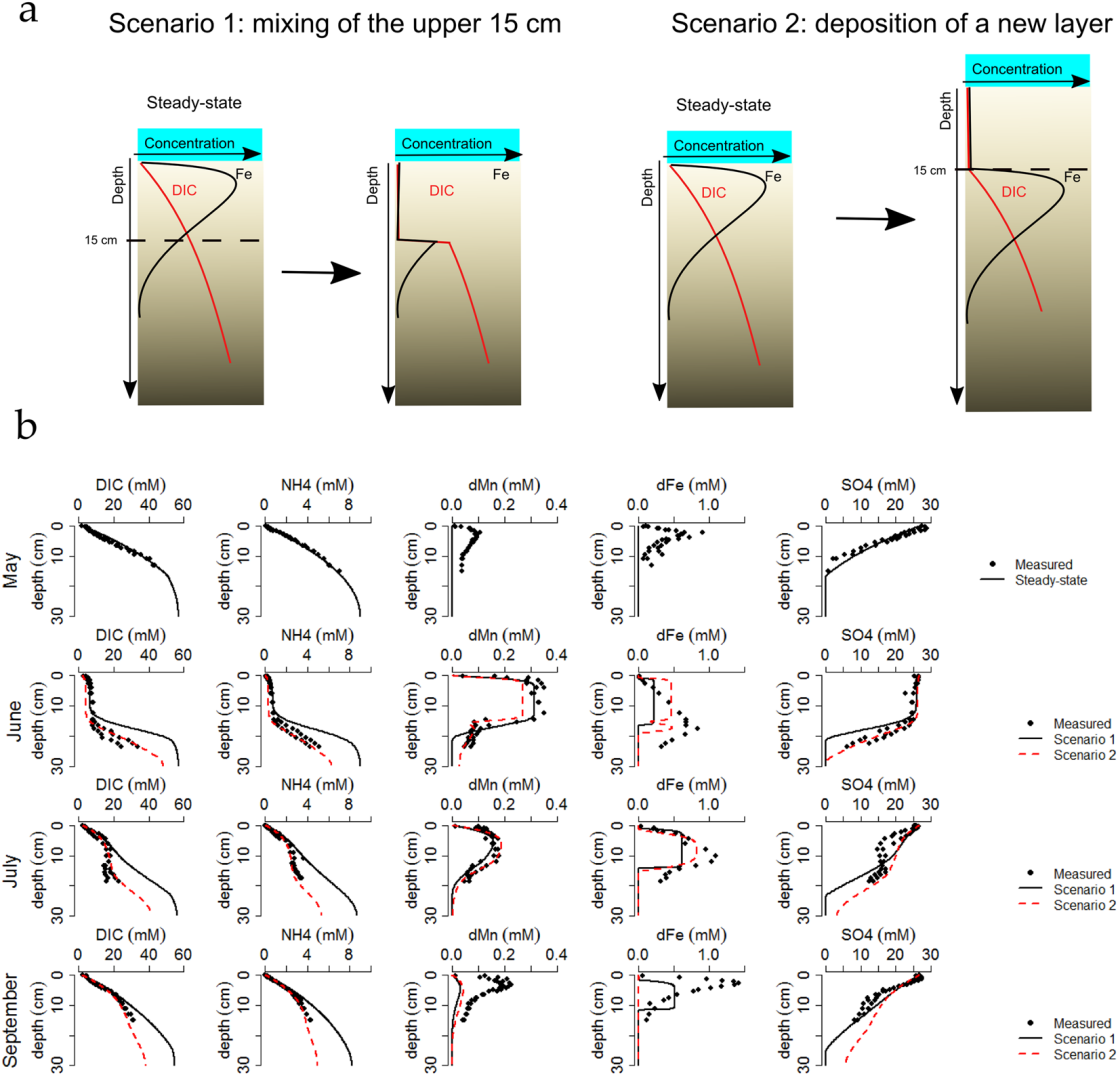
(**a**) Schematic representation of the two modelled scenarios (Scenario 1: bottom trawling, black full line – Scenario 2: deposition of 15 cm sediment, red dashed line). (**b**) Model fits to the measured data for the two scenarios.

To evaluate whether the sediment disturbance was bottom trawling or dredging related, we simulated the response of the sediment geochemistry using a transient diagenetic model (see Supplementary Information for details). In Scenario 1 (bottom trawling), we assume that the upper 15 cm of sediment is homogenised and the pore water is flushed with overlying water. The pore-water concentrations of the upper 15 cm are thus reset to their values in the overlying water. In Scenario 2 (disposal deposit), a new sediment layer of 15 cm is introduced on top of the existing sediment column. Again, the pore-water concentrations in the new layer are set to the overlying water values, but the original steady-state concentrations are preserved in the pore water below the new sediment deposit (Fig. 2a illustrates the difference in initial conditions between the two model scenarios).

For the model simulations to match the data, we had to assume in both scenarios that the upper 15 cm of sediment is enriched with fresh iron and manganese (oxyhydr)oxides upon disturbance. In Scenario 1, reduced iron and manganese minerals are exposed to oxygen during resuspension, which leads to re-oxidation and formation of oxidised iron and manganese minerals. If the new layer originates from a moving fluid mud layer (Scenario 2), the settling sediment is also exposed to oxygenated water, which hence also leads to the formation of iron and manganese oxy(hydr)oxides. We introduced constant concentrations of MnO_2_ and FeOOH in the upper 15 cm, and tuned the simulated concentrations to the observed pore-water trends. The additional input of reactive MnO_2_ was 1 µmol g^−1^, which corresponds to ~10% of the total solid phase manganese pool (Table S6), and is low compared to the solid phase manganese in sediments where an active manganese cycle is sustained (generally around 40–1000 µmol g^−1^)^46^. The additional input of FeOOH was 11 µmol g^−1^. A previous study at the field site has shown that there is about 150 µmol g^−1^ of reactive Fe^2+^ (extracted with 0.5 M HCl) in the sediments^41^, which would mean that ~7% of all reduced solid phase iron in the upper 15 cm of the sediment was oxygenated upon resuspension.

Furthermore, to match the transient recovery of mineralisation end-products (DIC and NH_4_^+^), we had to assume in both scenarios an input of fresh organic matter within the top 15 cm (2.5 mol C m^−2^ with a decay constant of 10 yr^−1^), which increased the existing inventory of organic carbon by only 2.2%. Such a (small) increase in overall organic carbon content is below the detection capabilities of current analytical techniques. Our model results suggest that this input of reactive carbon resulted in a short-term spike in the mineralisation rate, which initially increased to a peak value of 82 mmol m^−2^ d^−1^, after which it rapidly decreased with time (50 mmol m^−2^ d^−1^ after 20 days, dropping to 35 mmol m^−2^ d^−1^ after 80 days, which is still higher than the mineralisation rate of 16 mmol m^−2^ d^−1^ prior to the disturbance).

Both scenarios are able to adequately reproduce (Fig. 2) the transient recovery of the mineralisation end-products (DIC and NH_4_^+^), as well as the sequential utilisation of electron acceptors (manganese reduction, dissimilatory iron reduction and organoclastic sulphate reduction). In both cases, the dFe profiles show the least good fit, which is most likely caused by the simplified kinetics for iron adsorption (which is now included as an instantaneous equilibrium, see SI 1.2). Scenario 1 (bottom trawling) overestimates the concentrations of DIC, NH_4_^+^ and PO_4_^3−^ at depth, and the pore-water inventories are consistently too high during the recovery period (Fig. 2b). In contrast, Scenario 2 (disposal deposit) adequately matches the observed pore-water profiles; as such it appears that the disposal scenario is the most likely.

### Impact on organic matter mineralisation

Trawling and dredging of the seafloor causes the translocation, resuspension and deposition of considerable volumes of sediment. Our field data and modelling results reveal that such anthropogenically-steered resuspension/deposition events induce a transient biogeochemical cycling, which has the potential to substantially alter the organic carbon cycling within the seafloor.

Our model simulations suggest that the appearance of the >15 cm thick disturbed sediment layer was associated with a sudden and temporary increase in the mineralisation rate of organic matter. To align the model predicted depth profiles of DIC and NH_4_^+^ with their observed accumulation in the pore water, the model required a stepwise increase in the mineralisation rate via the introduction of a new pool of reactive organic matter. During the first 80 days of the recovery, the simulated average organic matter mineralisation rate was increased by a factor 2.5, from 16.4 mmol C m^−2^ d^−1^ to 41 mmol C m^−2^ d^−1^. Such enhanced mineralisation could be caused by different mechanisms. During the resuspension of the sediment, previously buried, refractory organic matter can be mixed with the fresh, fast degradable organic matter of the upper layers. This can lead to an increase in mineralisation^47^ through self-priming, in which more refractory compounds are broken down via co-mineralisation with fresh organic matter^48,49^. Incubation experiments have shown that mixing of fresh phytoplankton with old organic matter can lead to a priming effect of 30–60% (i.e. an increase in the background mineralisation on top of the increase caused by the addition of fresh organic matter)^50^. In our case, this would lead to a priming effect of ~5–10 mmol m^−2^ d^−1^ or 20–40% of observed increase in the mineralisation rate. Alternatively, phytoplankton in the water column can be entrained by settling sediment particles, and this leads to the input of fresh organic matter in the sediment column (phytoplankton trapping). During the first 80 days, a total of 1.2–1.6 mol C m^−2^ is additionally mineralised (excluding the background mineralisation and priming effect), which would correspond to 9–12% or 8–11 days of the spring primary production in the North Sea (estimated at 13 mol C m^−2^ – ref.^51^). Even though ‘phytoplankton trapping’ and ‘self-priming’ are plausible, there are other possible explanations for the transient increase in net mineralisation. The re-exposure of previously buried organic matter to oxygen can initiate decomposition of organic carbon that otherwise would remain refractory under continuous anaerobic conditions (e.g. as a result of oxidative cleavage of polymeric compounds)^49^. Alternatively, the promotion of iron and manganese redox cycling can also facilitate oxidation of refractory compounds (e.g. aromatic molecules)^52^. Most likely, multiple mechanisms were simultaneously active.

It should be noted that this increased mineralisation spike is not readily detectable with traditional flux techniques used in sediment geochemistry, such as O_2_ microsensor profiling and benthic flux chambers^53^. This is because the mineralisation spike leads to a non-steady state removal of electron acceptors (O_2_, NO_3_^−^) as well as a transient accumulation of mineralisation end-products (NH_4_^+^, DIC) in the pore water, which not necessarily affects the fluxes at the sediment-water interface. In the transient diagenesis event recorded here, most of the reduced species produced by carbon mineralisation (Mn^2+^, Fe^2+^, HS^−^) are removed by precipitation (as carbonates or sulphides) and do not contribute to the oxygen consumption. Therefore, the oxygen consumption rate of the sediment will be considerably smaller than the carbon mineralisation rate.

### Transient mineralisation pathways

**Figure 3:**
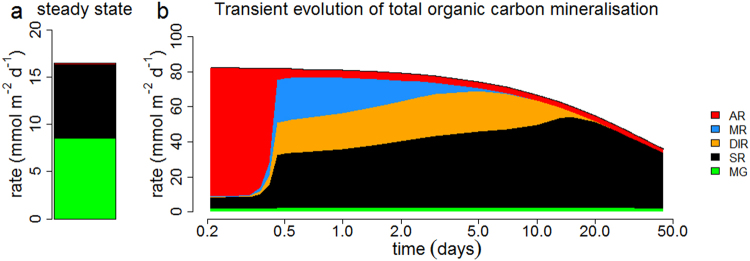
(**a**) Steady state of total organic carbon mineralisation rate and partitioning of the mineralisation pathways. (**b**) Transient evolution of the total organic carbon mineralisation rate and the relative importance of carbon mineralisation pathways. AR = aerobic respiration, MR = manganese reduction, DIR = dissimilatory iron reduction, SR = organoclastic sulphate reduction, MG = methanogenesis. Starting point of graph (**b**) is 5 hours after the event, note that the x-axis of panel b is in log-scale and that the y-axes have a different scale.

Sediment homogenisation events result in the flushing of the pore water with energetically favourable electron acceptors (O_2_, NO_3_^−^) and the injection of oxidised mineral electron acceptors (manganese and iron oxides) into deeper sediment horizons. This sudden increase in oxidising power has an immediate impact on the spatial partitioning of electron acceptors (Fig. 3). Before the disturbance event, both organoclastic sulphate reduction (47.5%) and methanogenesis (51.9%) were the dominant mineralisation pathways (integrated over 30 cm of sediment), while the contribution from aerobic respiration (0.6%) was negligible and no metal reduction was occurring (Fig. 3a). During the first 10 days of the recovery, there is a rapid alternation of electron acceptors (O_2_, MnO_2_ and FeOOH), which become sequentially depleted. After that, organoclastic sulphate reduction becomes the dominant mineralisation pathway for the next ~5 months (Fig. 3b). This follows the classical redox sequence of early diagenesis^38^, and a similar transient recovery has been observed in laboratory experiments where a fresh sediment layer (~7 cm) was deposited on top of incubated sediment cores^54^.

Just after the disturbance event, our model calculations show that the contribution of organoclastic sulphate reduction (47.5% to 8.1%) and methanogenesis (51% to 0.8%) sharply drops, while aerobic respiration is responsible for >90% of the depth-integrated organic matter mineralisation (OMM). This aerobic phase only lasts for a short time. Over the next 12 hours, the contribution of aerobic respiration (AR) decreases to 3%, due to the rapid depletion of oxygen in the sediment pore water. Manganese reduction peaks at 30% of the OMM after 2 days, but also rapidly disappears thereafter, as the small amount of freshly introduced manganese oxides becomes exhausted. The slow precipitation kinetics of manganese with calcite allows dissolved manganese to persist in the pore water for several weeks after manganese reduction ends. At a rate of ~0.72 mmol m^−2^ d^−1^ ref.^55^, it would take ~64 days to deplete the pore-water dMn inventory of 46 mmol m^−2^ as observed in May (Table S1), which closely agrees with the depletion time scale as simulated by the model (Fig. 2b). Dissimilatory iron reduction peaks at 31% of OMM after 3 days, just after the demise of manganese reduction, and its OMM contribution gradually diminishes over the next 12 days (Fig. 3b). Organoclastic sulphate reduction hence rapidly becomes the dominant electron acceptor after the disturbance event, and reaches a maximum of 90% of OMM after the disappearance of manganese and iron oxy(hydr)oxides from the sediment. Intriguingly, this is far above the initial steady state value of 47.5%. At the same time, methanogenesis was suppressed over the next 5 months and stayed well below the pre-disturbance steady state value of 51.9% of OMM. This shows that, even after 5 months, the sediment has not yet fully recovered from the impact and still resides in a transient state. The observed time-scales in mineralisation pathway recovery depend on two factors; (i) depletion of the electron acceptor and (ii) dynamics of the microbial community. It should be noted that the biogeochemical model here does not explicitly account for the colonisation and population dynamics of the resident microbial community, as is the common approach in early diagenetic modelling. In essence, kinetic rates in early diagenetic models are entirely controlled by substrate availability and not by the biomass of the microbial group that catalyses the reaction at hand. So, these models hence assume that microbial population instantaneously adapts to the substrate availability. The rapid response time of aerobic respiration (12 hours), manganese oxide reduction (2 days) and dissimilatory iron reduction (12 days), however, suggest that colonisation effects either are small or that the microbial populations have the ability to very quickly adapt.

The sudden exchange of the pore water in the upper 15 cm with the overlying water ([SO_4_^2−^] = 26 mM), brings in a substantial amount of sulphate, which allows sulphate concentrations at depth to remain high after 5 months (Fig. 1j). Assuming a contribution of organoclastic sulphate reduction of ~90% to OMM, a period of around 150 days is required to deplete the sulphate pore-water inventory of 2.6 mol S m^−2^ below 11 cm in June (the previous sulphate – methane transition zone). Note that this is a lower bound estimate, as this assumes that no sulphate is supplied via diffusion from the overlying water.

### Synthesis

The dataset and model simulations presented here illustrate how anthropogenically- induced sediment disturbance can change the carbon cycling and mineralisation pathways of organic matter in a cohesive coastal sediment. The principal driver for the observed shift in biogeochemical cycling is a sudden input of “reducing power” associated with the increased availability of reactive organic matter as well as the sudden input of “oxidising power”, associated with the *de novo* introduction of soluble and solid electron acceptors within the disturbed sediment horizon^54^. Effectively, the transient geochemistry that we observed is highly similar to what naturally occurs in the so-called ‘mobile deltaic muds’ of the Amazon delta^56^, where a combination of strong rotary tidal currents and wind-driven surface currents give rise to a regular (4 times every 24 hours) resuspension-deposition cycle. In each cycle, a mud package of 10–100 cm thick is homogenised, and from a biogeochemical perspective, these natural resuspension-deposition cycles induce similar responses as those associated with dredging/disposal and bottom trawling. The high disturbance frequency (up to 4 times per day) in the Amazon keeps the sedimentary system in a perpetual transient state^26,57^. Although anthropogenic disturbance typically occurs at substantially lower frequencies (e.g. 98.5% of the European coastal waters falls within the range of 0.02 to 4.85 yr^−1^)^3^, it affects the organic matter mineralisation pathways and sedimentary biogeochemical cycling in a comparable way.

**Figure 4:**
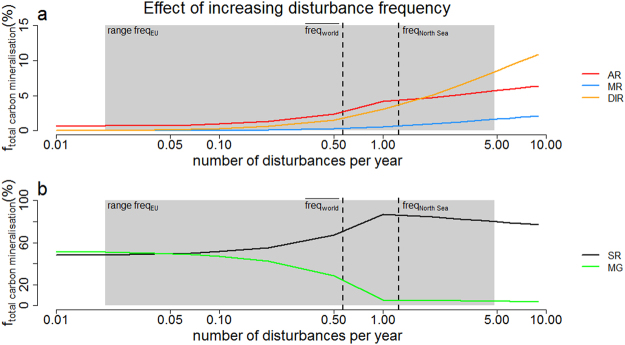
Effect of increasing disturbance frequency on the relative importance of the mineralisation pathways at field site BCZ130. y-axis is in % of the total integrated carbon mineralisation of the sediment column (30 cm) over a whole year. The grey area is the range of bottom trawling frequencies in European coastal waters (ref.^3^), $fre{q}_{NorthSea}$ indicates the bottom trawling frequency of the North Sea (ref.^3^) and $\overline{fre{q}_{world}}$ the estimated global average bottom trawling frequency of the continental shelves (ref.^60^). The x-axis is in log scale. AR = aerobic respiration, MR = manganese reduction, DIR = dissimilatory iron reduction, SR = organoclastic sulphate reduction, MG = methanogenesis.

The pore-water profiles within the mobile deltaic muds on the Amazon shelf exhibit very similar features as those shown in Fig. 1, with high concentrations of dFe (~0.5 mM) and dMn (~0.2 mM) and near constant SO_4_^2−^ profiles^57^. Moreover, these continuous resuspension-deposition cycles promote aerobic (oxygen reduction, nitrification) and metal oxide reduction (dissimilatory iron reduction and manganese reduction) mineralisation processes over anoxic processes^26^, which fully aligns with the observations here (Figs 3 and 4). The active reworking of the sediment on the Amazon shelf also stimulates mineralisation, decreases the sediment organic carbon content, and hence reduces the burial of organic carbon. Less than 30–35% of the riverine organic carbon flux (which is considered to be highly refractory) is buried within Amazon shelf sediments^26^. Similarly, our results here indicate that organic matter mineralisation is stimulated after sediment disturbance, which is likely - at least partly - due to the enhanced decomposition of previously buried refractory organic matter.

Figure 4 depicts the relative contribution of organic matter mineralisation pathways at our field site as a function of the disturbance frequency. At disturbance frequencies ranging from 1 to 5 yr^−1^ (as frequently observed in coastal waters with depths <400 m)^3,58^, aerobic respiration (4.2% to 5.7% of OMM), dissimilatory manganese reduction (0.6 to 1.6%), and dissimilatory iron reduction (3.1 to 8.6%.) are stimulated at the expense of organoclastic sulphate reduction. As trawling and dredging frequencies continue to increase, driven by increasing socio-economic needs, organoclastic sulphate reduction and methanogenesis will be increasingly suppressed, while aerobic respiration and metal oxide reduction will gain in importance.

The impact of anthropogenic reworking on the biogeochemistry of unbioturbated, muddy coastal sites bears some parallels with the historical development of agricultural land usage. Increases in agriculture land usage have led to a substantial decline in soil organic carbon content^59^, as the regular ploughing of crop fields stimulates organic matter degradation. We suggest here that a similar mechanism could be at work in coastal sites that are similar to our field site BCZ130. Our results show that, following a disturbance event, the organic carbon mineralisation is transiently stimulated, which over the longer term, will translate into reduced organic carbon burial rates. While this is potentially important (today ~50% of the total organic carbon burial takes place in cohesive coastal sediments like BCZ130)^19^, it is important to note that the seafloor is heterogenous, and most shelf sediments are either relict sands that do not accumulate organic carbon, or already experience a natural form of sediment mixing (e.g. bioturbation). More work is thus required to better constrain the full anthropogenic impact on the carbon cycle in the coastal ocean. However, our results provide a clear illustration of how human activities can inadvertently change the coastal carbon cycle.

### Data availability

The datasets generated during and/or analysed during the current study are available from the corresponding author on reasonable request.

## Electronic supplementary material

Supplementary information
