# Supplementary material for: Anthropogenic disturbance keeps the coastal seafloor biogeochemistry in a transient state
Source: Sci Rep. 2018 Apr 3;8:5582. doi: 10.1038/s41598-018-23925-y (PMC5883055; doi:10.1038/s41598-018-23925-y)
Supplement: Supplementary file 1 — Supplementary information [file 41598_2018_23925_MOESM1_ESM.pdf]

Supplementary information to – ‘Anthropogenic disturbance keeps the coastal seafloor biogeochemistry in a transient state’

Sebastiaan van de Velde [1,*]

Vera Van Lancker [2]

Silvia Hidalgo-Martinez [3]

William M. Berelson [4]

Filip J. R. Meysman [3,1]

[1] Analytical, Environmental and Geo-Chemistry, Vrije Universiteit Brussel, 1050 Brussel, Belgium

[2] Royal Belgian Institute of Natural Sciences. Operational Directorate Natural Environment, 1200 Brussel, Belgium

[3] Ecosystem Management Research Group, Department of Biology, Universiteit Antwerpen, 2160 Antwerpen, Belgium

[4] Earth Science Department, University of Southern California, CA 90089-0740 Los Angeles, USA

[*] Corresponding author: sebastiaan.van.de.velde@vub.be (Tel. +32 2 629 27 16)

Number of pages: S1 – S29

Number of tables: 8

Number of figures: 10

1. Supplementary info - METHODS

1.1 Solid phase analysis and water content

Water content was estimated from the same samples by weighing the empty centrifuge tubes, the centrifuge tubes filled with wet sediment and the tubes with sediment after freeze drying. Sediment porosity (volume of pore water per total volume of sediment) was determined from water content and solid phase density measurements, accounting for the salt content of the pore water. The solid phase density was determined by adding a known mass of grinded, freeze-dried sediment to a 100 ml graduated cylinder filled with water and recording the volume displacement.

Freeze-dried sediment samples were analyzed for particulate organic carbon (POC), particulate inorganic carbon (PIC) and particulate nitrogen (PN) by an Interscience Flash 2000 organic element analyzer. Samples for POC were acidified before analysis with 0.1N HCl to remove the inorganic carbon, and PIC was subsequently calculated by the difference between total carbon and POC[1]. Concentrations of POC, PIC and PN are expressed as mass % of dry sediment.

To determine the solid phase content of iron and manganese, freeze-dried sediment samples were ground to a fine powder for microwave assisted digestion (CEM Mars 5) using aqua regia as extraction agent (ISO 12914:2012). Subsequently, samples were analysed by High Resolution - Inductively Coupled Plasma – Mass Spectroscopy (HR-ICP-MS, ThermoScientific Element 2) after 100x dilution with Milli-Q water. Indium (2.5 ppb) containing 2% HNO_3 was injected simultaneously with the samples as an internal standard.

1.2 Diagenetic model formulation

The applied reactive transport model is an adaptation of the early diagenetic model used by van de Velde et al.[2] The mass balance equations are representative for a cohesive marine sediment without advection and bioturbation[3,4]

$$\begin{cases} \varphi \dfrac{\partial C_i}{\partial t} = \dfrac{\partial}{\partial z}\left(\varphi D_i \dfrac{\partial C_i}{\partial z} - \varphi v C_i \right) + \sum_k \nu_{i,k} R_k \\ (1-\varphi)\dfrac{\partial S_i}{\partial t} = \dfrac{\partial}{\partial z}\left(-(1-\varphi) w S_i \right) + \sum_k \nu_{i,k} R_k \end{cases} \quad [1]$$

where C_i represents the concentration of a solute in the pore water, S_i is the concentration of a solid component, z is the depth into the sediment and φ the porosity. R_k represents the reaction rate of the k-th reaction in the reaction set and $\nu_{i,k}$ denotes the stoichiometric

coefficient of the i-th species in the k-th reaction. In this model, only 2 transport processes are included (characteristic for a non-permeable sediment without macro-fauna): (i) solute diffusion in the pore water, by Fick's first law[5] (diffusion coefficient D_i) and (ii) downward advection due to sediment accumulation (advective velocities ν and *w*). where the flux is represented

$$J_D = -\varphi D_i \frac{\partial C}{\partial z} + \varphi v C \quad [2]$$

The reactive transport model includes 13 state variables in total; fast degradable organic matter [CH_2O]$_f$, manganese oxide [MnO_2], iron (oxyhydr)oxide [FeOOH], iron sulphide [FeS], manganese carbonate [$MnCO_3$], dissolved inorganic carbon [HCO_3^-], ammonium [NH_4^+], oxygen [O_2], sulphate [SO_4^{2-}], reduced manganese [Mn^{2+}], reduced iron [Fe^{2+}], sulphide [HS^-] and methane [CH_4]. Slowly degradable and refractory fractions of organic carbon are not modelled explicitly because previous field studies have shown that the organic carbon content of the field site does not vary appreciably over the time scale of several months[6,7]. The exclusion of these fractions considerably decreases model calculation time.

The solution procedure has been described previously[2,8], but in short, the open-source programming language R was used to implement a numerical solution procedure for the partial differential equations (Eq. [1]) by applying the method-of-lines[9] using the R packages CRAN:ReacTran[8] and CRAN:deSolve[10]. The sediment grid was generated by dividing the sediment domain (30 cm thickness) into an uneven grid of 400 layers with the thickness of the first layer being 0.0075 cm and the thickness of the other layers increasing with a factor 1.009.

Biogeochemical reaction set

The aim of the model simulations was to reproduce the disturbance event between May and June 2014, in order to estimate how organic matter mineralisation pathways evolve through time. The reaction set included (n=14) was tailored to this (Table S2).

Organic matter consists of three fractions; fast degradable organic matter (OC_f), slow degradable organic matter (OC_s) and refractory organic matter (OC_r). Refractory organic matter is considered to be conservative, i.e. it is not degraded over the time scale of the model. For the two other fractions, five mineralization pathways are included: aerobic respiration (AR), manganese reduction (MR), dissimilatory iron reduction (DIR), sulphate reduction (SR) and methanogenesis (MG). Denitrification is not included, as this is generally a negligible mineralization pathway[11], and measured rates are < 4 µmol N m^{-2} d^{-1} in the North

Sea[12]. The sequential usage of electron acceptors is based on thermodynamic free energy gain (AR > MR > DIR ≈ SR > MG) and implemented via the conventional limitation-inhibition formulation[13] (Table A 2). We did not include sulphate reduction inhibition by iron oxides, as recent studies have shown that both sulphate reduction and dissimilatory iron reduction can proceed simultaneously[2,14].

Oxidation of organic matter produces dissolved inorganic carbon (modelled as HCO_3^-) and ammonium (NH_4^+). Ammonium can subsequently adsorb onto solid phase particles[15] ($K_{ads}^{NH_4^+}$ = 1.75 cm^3 g^{-1}). The reduction of manganese oxides releases dissolved manganese (Mn^{2+}) which can adsorb onto solid phase particles[16] ($K_{ads}^{Mn^{2+}}$ = 30 cm^3 g^{-1}), or precipitate as manganese carbonate[16,17]. Aerobic manganese oxidation and aerobic ammonium oxidation were not included, as these process are generally slow[11] and will be of minor importance for the purpose of the model. In a similar fashion, DIR releases ferrous iron (Fe^{2+}), which can (i) adsorb onto solid phase particles[16,18], (ii) become reoxidised by oxygen, or (iii) precipitate as iron sulphide. Sulphate reduction produces free sulphide, which can be (i) reoxidised by oxygen (ii) reoxidised by iron (oxyhydr)oxides or (iii) precipitate as iron sulphide. Methanogenesis produces methane (CH_4) which can be oxidised by oxygen, iron oxides or sulphate (Table S2).

The adsorption of Fe^{2+}, NH_4^+, Mn^{2+} are included as a reversible, linear adsorption process (Table S2), where the concentration of the adsorbed species is in equilibrium at all times with the surrounding pore water, e.g., $\left[X \equiv Fe^{2+}\right] = K_{ads}^{Fe^{2+}} \left[Fe^{2+}\right]$, where $K_{ads}^{Fe^{2+}}$ is the adsorption constant (in cm^3 g^{-1}). This equilibrium formulation essentially assumes that adsorption proceeds much faster than the kinetics of the other reactions[16].

The kinetic rate expressions of all secondary redox reactions are described by standard second-order rate laws[3]. The kinetics of dissolution and precipitation of FeS and $MnCO_3$ also follow the standard rate laws, where the reaction rates are dependent on the pore water saturation state[17,19] (Table S3). The pH of the pore water is a controlling factor in the precipitation of FeS, but it is not explicitly modelled, in order to reduce model complexity. Instead, a constant depth profile with pH = 7.5 was adopted.

Model parametrisation

All kinetic parameters were taken from previous model studies and experimental studies[2,16,17,19–24] (Table S4). Upper boundary conditions for the solutes were set to a fixed concentration, based on the values measured in this and previous field campaigns[7] (Table S5). Upper boundary conditions for the solids were all set at a zero input flux, assuming that there was no input of iron and manganese oxides during the period of recovery (which is a reasonable assumption, considering the absence of iron and manganese oxides during the preceding months[7]). Bottom boundary conditions for all state variables were set at zero gradients.

Two types of model simulation were carried: (1) a steady state simulation describing the steady situation before the disturbance event and (2) transient simulation that describe the development of the sediment chemistry after the disturbance event. Table S4 shows the overview of all parameters as used in the steady-state simulation (the month May). For the dynamic model simulation after the disturbance event (months June, July, September) one needs to consider that in reality, adsorption is not instantaneous. It is a dynamic process and not much is known about the dynamics of reversible adsorption in natural systems. Therefore, there is a lot of uncertainty on the rates of adsorption. Rather than modelling adsorption dynamically (which immensely increases model calculation time) we have reduced the equilibrium constants of ammonium, manganese and iron, which partially accounts for the dynamics of adsorption (slower adsorption leads to less adsorbed species vs dissolved species, which is essentially equivalent to a lower saturation constant). The steady-state simulation was run with the K-values given in Table S4, while for the dynamic simulation run, K-values were set at 1.75 $cm^3\ g^{-1}$, 5 $cm^3\ g^{-1}$ and 10 $cm^3\ g^{-1}$ for adsorbed ammonium, manganese and iron, respectively.

Steady state simulation

In a first step, the model was allowed to reach a steady state with (i) no iron and manganese oxides (ii) no fast degradable organic carbon (which is only brought in during the disturbance event) and (iii) a fixed concentration depth profile of (slow degradable and refractory) organic carbon of 2%. The fraction of slow degradable vs refractory organic matter was tuned to the DIC and SO_4^{2-} profiles. The adsorption coefficient and the C:N ratio of organic matter were tuned to the NH_4^+ profile. Resulting model profiles are represented in Figure A 1. Important to note is that this site is dominated by the electrogenic sulphur oxidation (e-SOx). This process was not included in the biogeochemical model, because (i) it

has little impact on the partitioning of the OMM pathways[7,25] and (ii) the understanding of e-SOx is limited to its steady state effect on sediment geochemistry, while the dynamics are still largely unkown[22]. The main effect of e-SOx on the sediment geochemistry is the strong release of Fe and Mn in the pore water due to the dissolution of sulphides and carbonates[26]. It has been shown at this field site that the release in the pore water of Fe and Mn is solely the result of e-SOx[7], and since e-SOx is not included in the model, no release of iron and manganese is witnessed in the pore water.

Dynamic simulation

In a second step, a disturbance was introduced. Figure 2a in the main text provides a schematic representation of the two scenarios: (1) a homogenisation of the upper 15 cm of sediment by trawling or (2) the deposition of a new layer of 15 cm on top of the sediment by dredging (see section 2 for details). The initial state before the disturbance was defined by modelled steady state profiles of DIC, NH_4^+ and SO_4^{2-}, together with measured pore water profiles of dissolved Fe and Mn in May 2014. In the trawling scenario, the upper 15 cm of the model domain were modified as follows: (i) the profiles of DIC, NH_4^+, PO_4^{3-} and SO_4^{2-} were set to their overlying water values (Table S5), (ii) the concentration of reduced species (dFe, dMn) were set to zero, simulating oxidation in the overlying water (iii) fast degradable organic carbon, iron and manganese oxides were newly introduced. In the dredging scenario, a new layer of 15 cm was added to the model domain, and this zone was treated as above. The only difference is thus the lower 15 cm of the modelled domain, were in scenario 1 the concentration profiles of the lower 15 cm of the steady state were taken, and in scenario 2 the upper 15 cm of the steady state. The model then calculated the transient evolution over the course of 78 days (which is equivalent with the evolution from June to September 2014). The amount of reactive organic carbon, iron and manganese oxides that was newly introduced was tuned to match the observed evolution of DIC, NH_4^+ (for the amount of reactive organic carbon) and dMn and dFe (for the amount of iron and manganese oxides).

2. Supplementary results

2.1 Sediment coloration

During sampling campaigns in spring (January, March and May 2014) the sediment appearance and coloration was described as clayish, with a dark grey colour and the occasional appearance of a millimetre thick sand layer[7] (Figure S3a). In contrast, the cores sampled in June 2014 (this campaign) were light brown in colour. In the following months, the sediment gradually changed colour, from brown to grey, and in September, the colour was similar to the months before June. The change in sediment coloration suggests that the overlying layer is first oxidised (the loss of the dark colour is possibly through the oxidation or loss of FeS minerals, while the brown colour is typical for Manganese oxides[2]) and then reduced over time (regaining of the compactness and dark colour).

2.2 Cohesiveness and porosity

The gravity corer in June penetrated much deeper in the sediment (up to 24 cm, as compared to 15 cm in May, Figure 1 in the main text), indicating that the sediment was less compact (Figure S3b). Over time, the penetration decreased again to ~15 cm.

The porosity profile in May showed scatter around a mean value of 0.79 in the upper few centimetres and decreased with depth from a maximum value of 0.82 to ~0.70 at a depth of 15 cm. In June, the porosity profile started at the same value at the sediment water interface (0.82), but remained constant at a value of 0.78 between 1 cm and 15 cm, and then decreased to a 0.68 at 24 cm. This suggests that the upper 15 cm of the sediment column in June was freshly deposited (without compaction) or thoroughly mixed. In July, porosity was higher at the SWI (~0.91) and decreased rapidly to 0.75, after which it recovered to 0.80. Below 11 cm it decreased to ~0.70. Possibly, the upper layer compacted between June and July, while the rest compacted between July and September, when the porosity profiles again resembled those of May (before the disturbance event), with some scatter and a decrease from ~0.85 to ~0.70 at a depth of 15 cm (Figure S8).

2.3 Elemental composition of solid phase sediment

Bottom trawling and sediment deposition after dredging may affect the elemental composition of the solid phase in a different way. In the case of bottom trawling, local sediment is resuspended, and deposited as a mixed layer. Hence one expects the upper mixed layer to be similar in the upper disturbed layer and the underlying undisturbed layer. In the case of deposition of dredged sediment, another type of sediment could be deposited, which may have a different chemical composition.

Figure A 6 shows the solid phase concentrations of particulate organic carbon (POC), particulate inorganic carbon (PIC), particulate nitrogen (PN) and aqua regia extractable iron and manganese, averaged per 5 cm interval and for two replicate cores. The mean values and standard deviations are presented in Table A5. The concentrations in the 5 cm layer in June were consistently higher than in May, for POC (~0.7%), PN (~0.11%), extractable Fe (~110 µmol g^{-1}) and extractable Mn (~5 µmol g^{-1}). In contrast, PIC was constant for all layers and for all months (Figure S9). However, the standard deviations are relatively big when compared to these differences. To check whether there was a statistically significant difference between the different months, a standard two-way ANOVA with replicate was performed with month and depth interval as factors (significance level $p = 0.05$). The only significant difference was found for PIC ($p = 0.03$, Table A 7). This suggests that most of the difference between June and May can be explained by spatial variability, rather than different sediment characteristics.

Similarly, trace metal concentrations (As, Cd, Cr, Cu, Pb, Ni and Zn; aqua regia extractable) is given in Figure S10 (mean values and standard deviations in Table S6). If the layer was a deposition of dredged sediment originating from navigation channels and/or harbours, one would expect that the solid phase of the sediment shows similar characteristics as the sediment in the harbour zones. Therefore, the trace metal content of the dredged material from the harbours in the Belgian Coastal Zone (dashed and filled line in Figure S10 and second and third column Table S7) are given as a reference for the measured concentrations. As for the major element concentrations, a two-way ANOVA was used to evaluate the apparent differences in trace metal content. Only lead and arsenic gave a significant result (Table S8). This shows as well that the high value in June is most likely not because of different sediment characteristics.

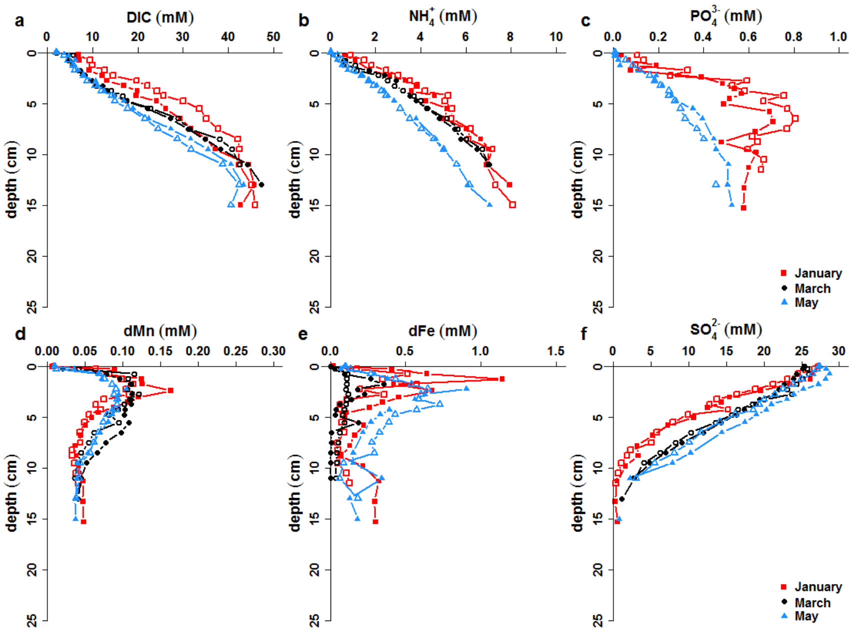

Figure S1: Pore water profiles of three months in the period before the disturbance: January 2014 (red), March 2014 (black) and May 2014 (blue). (a) dissolved inorganic carbon (DIC), (b) ammonium (NH_4^+), (c) phosphate (PO_4^{3-}), (d) dissolved manganese (dMn), (e) dissolved iron (dFe) and (f) sulphate (SO_4^{2-}).

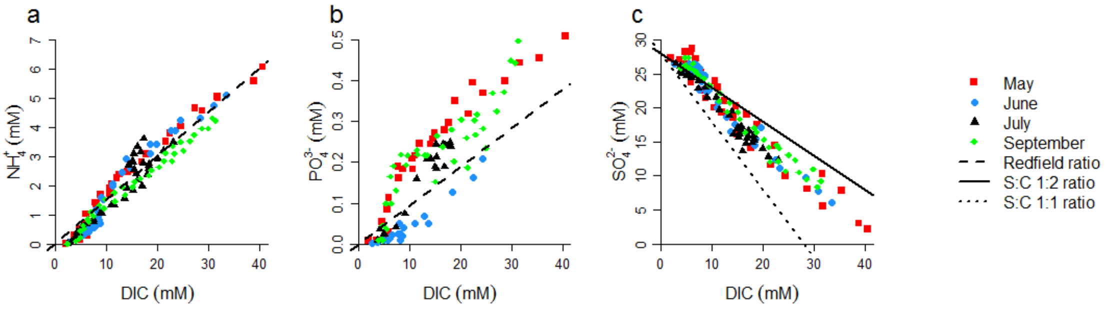

Figure S2: Correlations between the pore water concentrations of Dissolved Inorganic Carbon (DIC) and (a) ammonium (NH_4^+), (b) phosphate (PO_4^{3-}) and (c) sulphate (SO_4^{2-}) for the months May, June, July and September. The black dashed line in panel (a) and (b) indicates the Redfield ratio (C:N:P = 106:16:1), the black solid line in (c) shows the stoichiometric 1:2 (S:C) ratio for sulphate reduction and the black dotted line shows the stoichiometric 1:1 (S:C) ratio for anaerobic oxidation of methane.

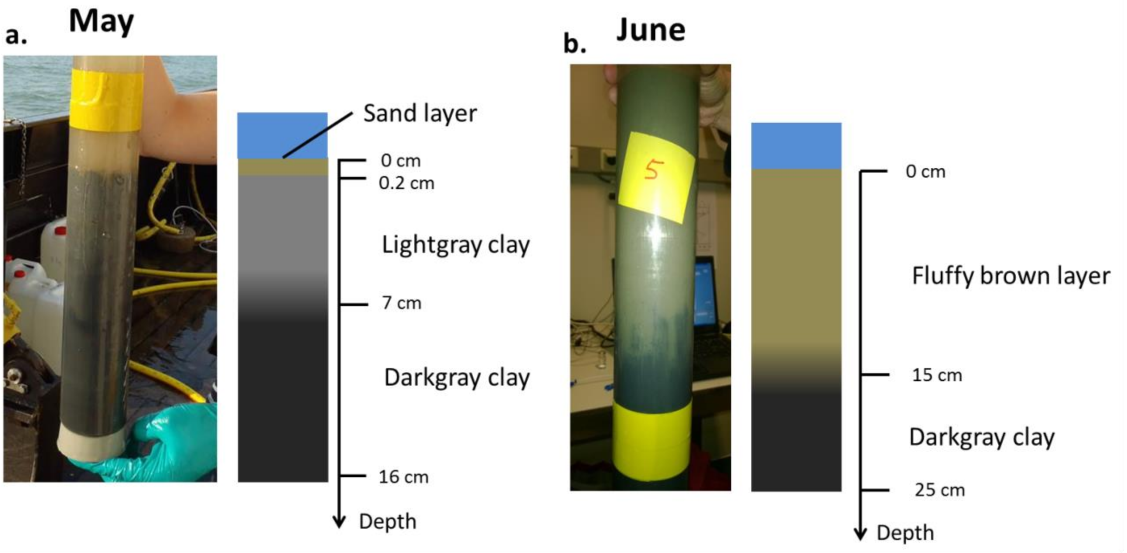

Figure S3: Photographic images of sediment cores immediately after core collection and a schematic description of the downward colour changes in the sediment. (a) May 2014, right before the disturbance event. The oxidized brownish layer is limited to the upper ~0.2 cm. (b) June 2014, after the disturbance event. The oxidized brownish layer now reaches down to ~16 cm. Note also the difference in core length. In May 2014, sediment cores collected were only ~16cm, while in June 2014, the gravity corer penetrated much deeper, and sediment cores collected were ~25cm.

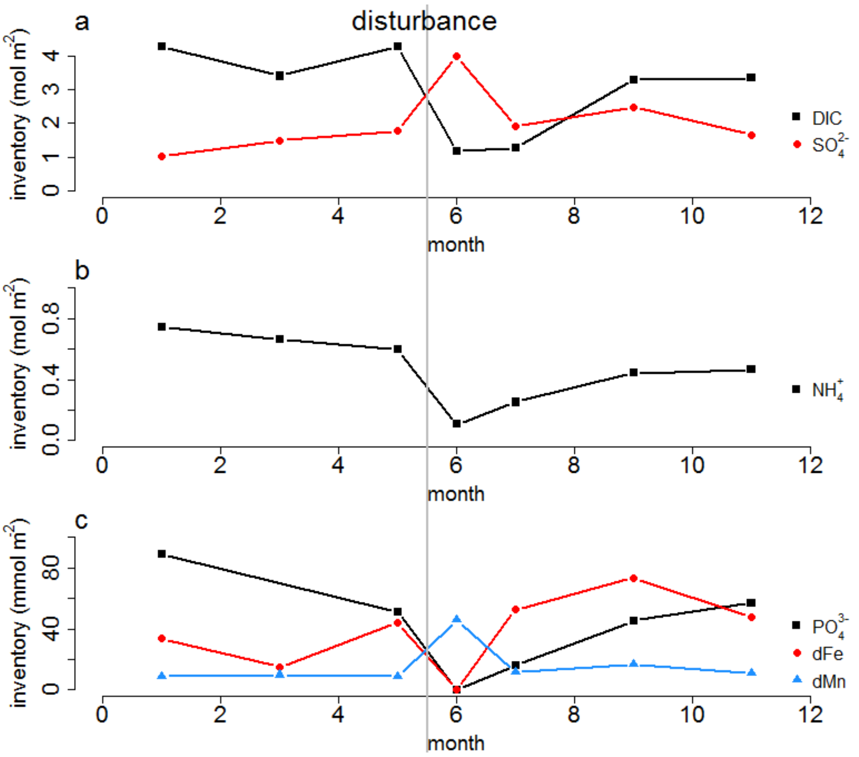

Figure S4: Evolution of the pore water inventories across 2014 (see Table S1 for exact values). Grey line indicates when the disturbance occurred. (a) Dissolved inorganic carbon (DIC) and sulphate (SO_4^{2-}), (b) ammonium (NH_4^+), (c) phosphate (PO_4^{3-}), dissolved iron (dFe) and dissolved manganese (dMn).

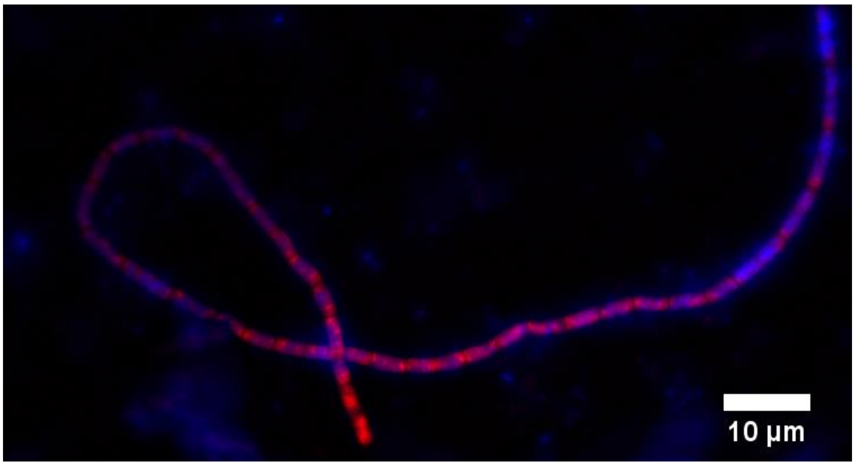

Figure S5: image obtained by Fluorescence In Situ Hybridization (probe DSB706) of a cable bacteria fragment from the September cores at the field site, providing evidence for the recolonization of cable bacteria. See ref. [25] for more information on the sampling procedure.

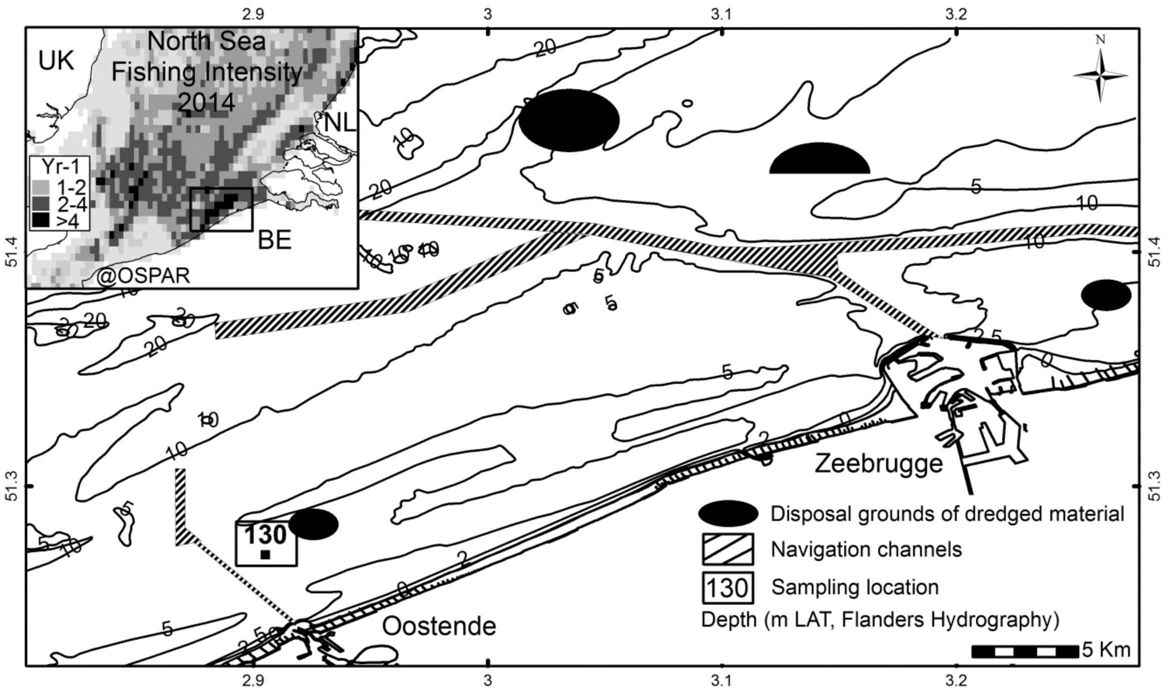

Figure S6: Dredging and trawling in the North Sea, with focus on the Belgian coastal zone. Fishing intensity (upper left) is indicated in swept-area ratio (mean number of times per year that each cell was traversed by fishing gear disturbing the subsurface (OSPAR 2014; https://odims.ospar.org)). Towards the harbours dredging occurs to maintain navigable depth. Dredged sediments are disposed of at designated disposal grounds. Sampling station 130 is situated between a navigation channel and a disposal ground. (LAT: Lowest Astronomical Tide). Data were compiled (ArcGIS10.3) using freely available datasets (https://odnature.naturalsciences.be/marine-atlas/data).

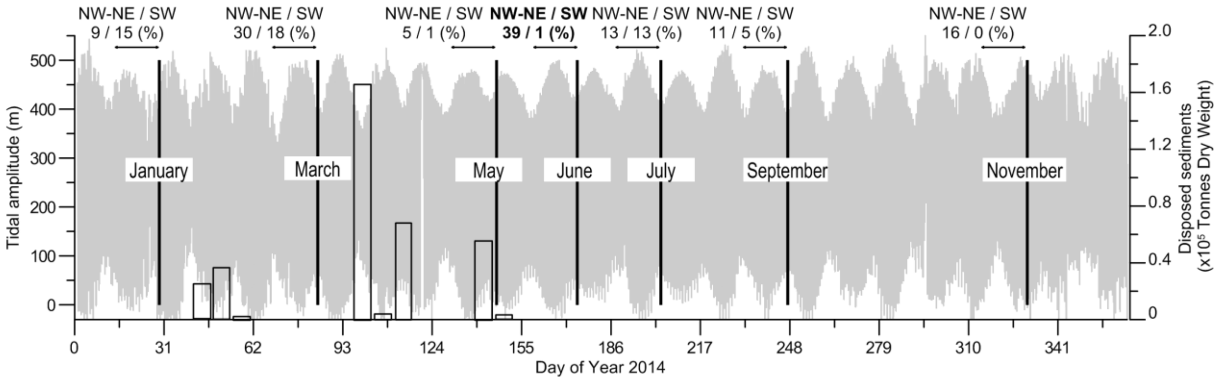

Figure S7: Hydro-meteorological conditions and dredging activities together with the sampling campaigns. Background (grey) is the tidal amplitude (m) showing the Spring-Neap tidal cycles throughout 2014. Significant wave height of > 1 m is given in frequency of occurrence (%) for the sectors NW-NE and SW, and is calculated over a 14-days period prior to the sampling. Dredged material was disposed at a disposal ground nearby, mostly in Spring (bars).

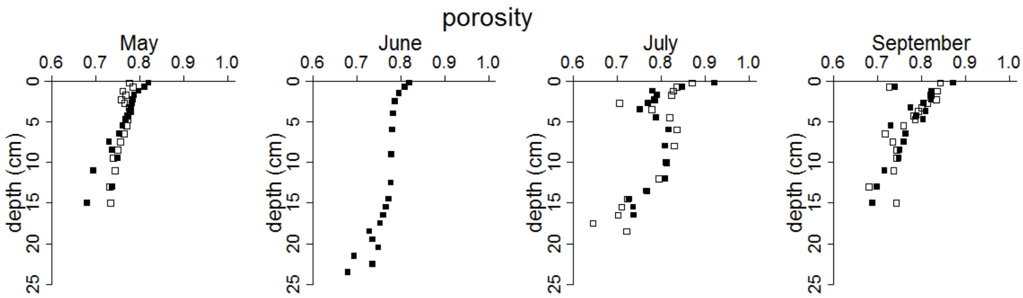

Figure S8: Porosity from duplicate cores from May, June, July and September 2014. In June only 1 core was measured for porosity.

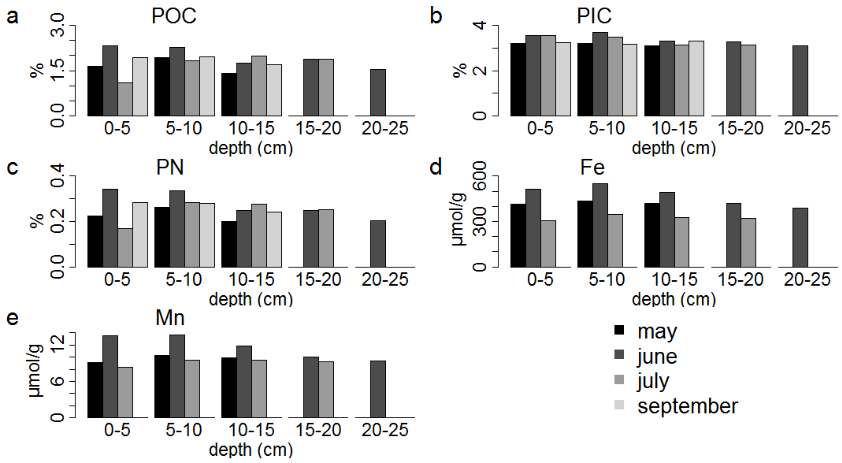

Figure S9: Comparison of the average elemental composition of the sediment cores. The content is averaged over 5 cm intervals. Concentrations of (a) POC, (b) PIC and (c) PN are in mass-%, concentrations of (d) aqua regia extractable Fe and (e) Mn are in µmol g^{-1}. Fe and Mn content where not measured in September.

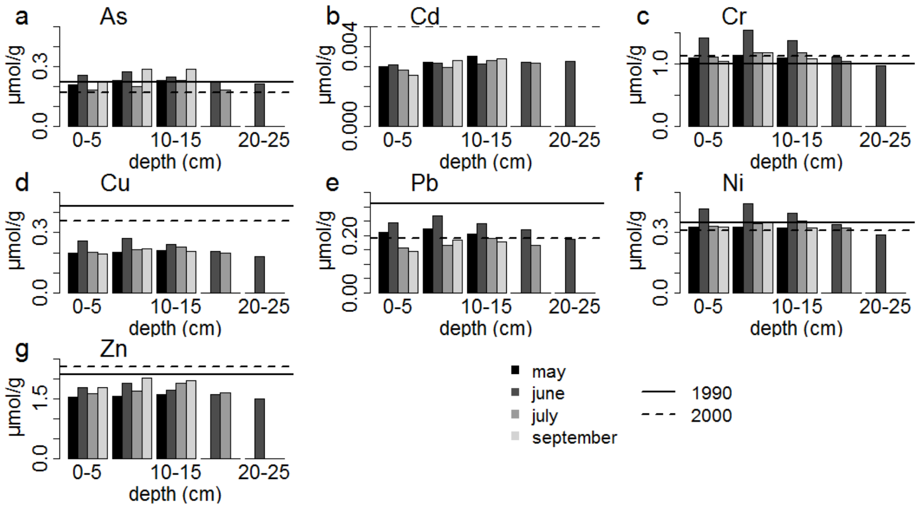

Figure S10: Comparison of the average aqua regia extracteable trace metal content of the sediment cores. (a) As, (b) Cd, (c) Cr, (d) Cu, (e) Pb, (f) Ni and (Zn). The dashed line is the mean trace element content in dredged sediment from the Oostende harbour in 2000, the full line in 1990 (http://www.mumm.ac.be/NL/Management/Sea-based/dredging.php). The Cd concentration in 1990 was much higher than the measured concentration in our samples (0.022 µmol g^{-1}). The content is averaged over 5 cm intervals. Concentrations are in µmol g-1. In September only 1 core was measured for As.

solute	month	inventory (mmol m^{-2})	solute	month	inventory (mmol m^{-2})
DIC	January	4263	dMn	January	8.9
	March	3394		March	9.8
	May	4276		May	8.9
	June	1190		June	46
	July	1281		July	12
	September	3289		September	17
	November	3348		November	11
NH_4^+	January	743	dFe	January	34
	March	663		March	15
	May	595		May	44
	June	105		June	0.07
	July	251		July	53
	September	442		September	74
	November	464		November	48
PO_4^{3-}	January	89	SO_4^{2-}	January	1039
	March	NA		March	1490
	May	51		May	1784
	June	0.015		June	3982
	July	16		July	1907
	September	45		September	2482
	November	57		November	1659

Table S1: Evolution of the pore water inventories of the 16 top cm for the different analytes. Inventory is calculated as $\sum_{x_{SWI}}^{x_{depth}} C_i \cdot \Delta x_{slice}$.

Primary redox reactions			
R1	Aerobic respiration	AR_s	$\{CH_2O.(NH_3)_{1/R_{CN}}\}_s + O_2 \rightarrow HCO_3^- + \frac{1}{R_{CN}} NH_4^+ + \frac{R_{CN}-1}{R_{CN}} H^+$
R2	Manganese reduction	MR_s	$\{CH_2O.(NH_3)_{1/R_{CN}}\}_s + 2MnO_2 + \frac{3R_{CN}+1}{R_{CN}} H^+ \rightarrow HCO_3^- + \frac{1}{R_{CN}} NH_4^+ + 2Mn^{2+} + 2H_2O$
R3	Dissimilatory iron reduction	DIR_s	$\{CH_2O.(NH_3)_{1/R_{CN}}\}_s + 4FeOOH + \frac{7R_{CN}+1}{R_{CN}} H^+ \rightarrow HCO_3^- + \frac{1}{R_{CN}} NH_4^+ + 4Fe^{2+} + 6H_2O$
R4	Sulphate reduction	SR_s	$\{CH_2O.(NH_3)_{1/R_{CN}}\}_s + \frac{1}{2} SO_4^{2-} \rightarrow HCO_3^- + \frac{1}{R_{CN}} NH_4^+ + \frac{1}{2} HS^- + \frac{R_{CN}-2}{2R_{CN}} H^+$
R5	methanogenesis	MG_s	$\{CH_2O.(NH_3)_{1/RCN}\}_s + \frac{3R_{CN}+2}{2R_{CN}} H^+ \rightarrow \frac{1}{2} HCO_3^- + \frac{1}{R_{CN}} NH_4^+ + \frac{1}{2} CH_4 + \frac{1}{2} H_2O$
Secondary redox reactions			
R6	Ferrous iron oxidation	FIO	$4Fe^{2+} + O_2 + 6H_2O \rightarrow 4FeOOH + 8H^+$
R7	Sorbed ferrous iron oxidation	SIO	$4X \equiv Fe^{2+} + O_2 + 6H_2O \rightarrow 4FeOOH + 8H^+$
R8	Canonical sulphur oxidation	CSO	$HS^- + 2O_2 \rightarrow SO_4^{2-} + H^+$
R9	Sulphide-mediated iron reduction	SIR	$HS^- + 8FeOOH + 15H^+ \rightarrow SO_4^{2-} + 8Fe^{2+} + 12H_2O$
R10	Aerobic methane oxidation	ArMO	$CH_4 + 2O_2 \rightarrow CO_2 + 2H_2O$
R11	Fe mediated methane oxidation	Fe-AMO	$CH_4 + 8FeOOH + 16H^+ \rightarrow CO_2 + 8Fe^{2+} + 14H_2O$
R12	Sulphate mediated methane oxidation	S-AMO	$CH_4 + SO_4^{2-} + H^+ \rightarrow CO_2 + HS^- + 2H_2O$
Sorption/precipitation reactions			
R13	Iron sulphide precipitation	ISP	$Fe^{2+} + HS^- \rightarrow FeS + H^+$
R14	Iron sulphide dissolution	ISD	$FeS + H^+ \rightarrow Fe^{2+} + HS^-$
R15	Manganese carbonate precipitation	MCP	$Mn^{2+} + HCO_3^- \rightarrow MnCO_3 + H^+$
R16	Manganese carbonate dissolution	MCD	$MnCO_3 + H^+ \rightarrow Mn^{2+} + HCO_3^-$
R17	Ammonium sorption	AMS	$NH_4^+ \leftrightarrow X \equiv NH_4^+$
R18	Manganese sorption	MnS	$Mn^{2+} \leftrightarrow X \equiv Mn^{2+}$
R14	Ferrous iron sorption	FIS	$Fe^{2+} \leftrightarrow X \equiv Fe^{2+}$

Table S2 List of biogeochemical reactions included in the reactive transport model. A distinction is made between mineralization reactions of organic matter ("Primary redox reactions"), the subsequent reoxidation of mineralization products ("Secondary redox reactions), and sorption and precipitation reactions ("Sorption/precipitation reactions"). The associated kinetic expressions are listed in Table 3. For the primary redox reactions only the reactions for **slow degradeable organic matter (CH2Os) are given. The reactions for fast degradable organic matter are identical.**

	Reaction	Kinetic rate expression
M1	Mineralization fast organic matter ($R_{\min}^{f}$)	$(1-\varphi)k_f\left[CH_2O_f\right]$
M2	Mineralization slow organic matter ($R_{\min}^{s}$)	$(1-\varphi)k_s\left[CH_2O_s\right]$
R1	Aerobic respiration	$\frac{[O_2]}{[O_2]+K_{O_2}}R_{\min}^{s}$
R2	Manganese reduction	$\frac{[MnO_2]}{[MnO_2]+K_{MnO_2}}\frac{K_{O_2}}{[O_2]+K_{O_2}}R_{\min}^{s}$
R3	Dissimilatory iron reduction	$\frac{[FeOOH]}{[FeOOH]+K_{FeOOH}}\frac{K_{O_2}}{[O_2]+K_{O_2}}\frac{K_{MnO_2}}{[MnO_2]+K_{MnO_2}}R_{\min}^{s}$
R5	Sulphate reduction	$\frac{[SO_4^{2-}]}{[SO_4^{2-}]+K_{SO_4^{2-}}}\frac{K_{O_2}}{[O_2]+K_{O_2}}\frac{K_{MnO_2}}{[MnO_2]+K_{MnO_2}}R_{\min}^{s}$
R7	Methanogenesis	$\frac{K_{SO_4^{2-}}}{[SO_4^{2-}]+K_{SO_4^{2-}}}\frac{K_{FeOOH}}{[FeOOH]+K_{FeOOH}}\frac{K_{MnO_2}}{[MnO_2]+K_{MnO_2}}\frac{K_{O_2}}{[O_2]+K_{O_2}}R_{\min}^{f}$
R8	Ferrous iron oxidation	$\varphi k_{FIO}[O_2][Fe^{2+}]$
R7	Sorbed ferrous iron oxidation	$(1-\varphi)k_{SIO}[O_2][X\equiv Fe^{2+}]$
R8	Canonical sulphur oxidation	$\varphi k_{CSO}[O_2][HS^-]$
R9	Sulphide-mediated iron reduction	$(1-\varphi)k_{SIR}[FeOOH][HS^-]$
R10	Aerobic methane oxidation	$\varphi k_{ArMO}[O_2][CH_4]$
R11	Iron mediated methane oxidation	$(1-\varphi)k_{Fe-AMO}[FeOOH][CH_4]$
R12	Sulphate mediated methane oxidation	$\varphi k_{S-AMO}[SO_4^{2-}][CH_4]$
R13	Iron sulphide precipitation	$(1-\varphi)k_{ISP}\left(\frac{[Fe^{2+}][HS^-]}{[H^+]K_{FeS}^{SP}}-1\right)^{n_{ISP}}$
R14	Iron sulphide dissolution	$(1-\varphi)k_{ISD}[FeS]\left(1-\frac{[Fe^{2+}][HS^-]}{[H^+]K_{FeS}^{SP}}\right)^{n_{ISD}}$
R15	Manganese carbonate precipitation	$(1-\varphi)k_{MCP}\left(\frac{[Mn^{2+}][HCO_3^-]}{[H^+]K_{MnCO_3}^{SP}}-1\right)^{n_{MCP}}$
R15	Manganese carbonate dissolution	$(1-\varphi)k_{MCD}[MnCO_3]\left(1-\frac{[Mn^{2+}][HCO_3^-]}{[H^+]K_{MnCO_3}^{SP}}\right)^{n_{MCD}}$

Table S3 List of kinetic rate expressions for the reactions included in the reactive transport model. All expressions are based on standard kinetic formulations used in sediment biogeochemical models (see text for details). The values of the kinetic constants are listed in Table S4. Only the full expressions for mineralisation of slow degradable organic matter are given, expressions for fast degradable are identical.

Constant	Symbol	Unit	Value	Reference
Organic matter reduction				
Decay constant fast degradeable organic matter	k_f	yr^{-1}	10	a
Decay constant slow degradeable organic matter	k_s	yr^{-1}	0.1	b
Monod constant oxygen consumption	K_{O_2}	$\mu mol\ cm^{-3}$	0.001	c,d
Monod constant manganese reduction	K_{MnO_2}	$\mu mol\ cm^{-3}$	2.6	e
Monod constant dissimilatory iron reduction	K_{FeOOH}	$\mu mol\ cm^{-3}$	10.4	e
Monod constant sulphate reduction	$K_{SO_4^{2-}}$	$\mu mol\ cm^{-3}$	0.9	d
Oxidation reactions				
Ferrous iron oxidation	k_{FIO}	$\mu mol^{-1}\ cm^3\ yr^{-1}$	10^{+7}	d
Sorbed ferrous iron oxidation	k_{SIO}	$\mu mol^{-1}\ cm^3\ yr^{-1}$	10^{+7}	f
Canonical sulphide oxidation	k_{CSO}	$\mu mol^{-1}\ cm^3\ yr^{-1}$	10^{+7}	d
Sulphide-mediated iron reduction (oxyhydr)oxides	k_{SIR}	$\mu mol^{-1}\ cm^3\ yr^{-1}$	494	g
Aerobic methane oxidation	k_{ArMO}	$\mu mol^{-1}\ cm^3\ yr^{-1}$	10^{+4}	e
Fe mediated methane oxidation	k_{Fe-AMO}	$\mu mol^{-1}\ cm^3\ yr^{-1}$	10^{+4}	-
Sulphate mediated methane oxidation	k_{S-AMO}	$\mu mol^{-1}\ cm^3\ yr^{-1}$	10	e
precipitation reactions				
Iron sulphide precipitation	k_{ISP}	$\mu mol^{-1}\ cm^3\ yr^{-1}$	10^{+4}	d
Iron sulphide dissolution	k_{ISD}	yr^{-1}	3	d
Kinetic exponent iron sulphide precipitation	n_{ISP}	-	1	d
Kinetic exponent iron sulphide dissolution	n_{ISD}	-	1	d
Manganese carbonate precipitation	k_{MCP}	$\mu mol^{-1}\ cm^3\ yr^{-1}$	$0.5*10^{-5}$	e
Manganese carbonate dissolution	k_{MCD}	yr^{-1}	0	e
Kinetic exponent Manganese carbonate precipitation	n_{ISP}	-	1	e
Kinetic exponent Manganese carbonate dissolution	n_{MCD}	-	1	e
Saturation constants				
Equilibrium constant ammonium sorption	$K_{ads}^{NH_4^+}$	$cm^3\ g^{-1}$	1.75	-
Equilibrium constant manganese sorption	$K_{ads}^{Mn^{2+}}$	$cm^3\ g^{-1}$	30	h
Equilibrium constant ferrous iron sorption	$K_{ads}^{Fe^{2+}}$	$cm^3\ g^{-1}$	696	h
Saturation constant iron sulphide	K_{FeS}^{SP}	$\mu mol\ cm^{-3}$	3160	i
Saturation constant manganese carbonate	$K_{MnCO_3}^{SP}$	$\mu mol\ cm^{-3}$	10^{-5}	e

Table S4 Parameter values for the kinetic constants included in the steady-state simulation of the reactive transport model. All parameters are expressed per bulk volume of sediment. References: a: (Katsev et al. 2006); b: (Fossing et al. 2004); c: (Van Cappellen and Wang 1996); d: (Meysman et al. 2015); e: (Meysman et al. 2003); f: (van de Velde and Meysman, 2016); g: (Poulton et al. 2004); h: (Berg et al. 2003); i: (Rickard 2006).

Parameter	Depth profile		Symbol	Unit	Value
Salinity	Constant		S	-	33
Temperature	Constant		T_C	°C	20
Pressure	Constant		P	bar	1.013
pH	Constant		pH	-	7.5
Porosity	Constant		φ	-	0.75
Solid phase density	Constant		ρ_{sed}	g cm^{-3}	2.6
Sedimentation velocity	Constant	porewater	v	cm yr^{-1}	0.2
		solid phase	w	cm yr^{-1}	0.2
C:N ratio organic carbon	Constant		R_{CN}	-	5
**Species**	**UBC**	**Type**	**Unit**		**Value**
$FeOOH$	FF	Solid	µmol cm^{-2} yr^{-1}		0
MnO_2	FF	Solid	µmol cm^{-2} yr^{-1}		0
FeS	FF	Solid	µmol cm^{-2} yr^{-1}		0
$MnCO_3$	FF	Solid	µmol cm^{-2} yr^{-1}		0
DIC	FC	solute	µmol cm^{-3}		2.2
NH_4^+	FC	solute	µmol cm^{-3}		0
O_2	FC	solute	µmol cm^{-3}		0.28
SO_4^{2-}	FC	solute	µmol cm^{-3}		26.6
Mn^{2+}	FC	solute	µmol cm^{-3}		0
Fe^{2+}	FC	solute	µmol cm^{-3}		0
HS^-	FC	solute	µmol cm^{-3}		0
CH_4	FC	solute	µmol cm^{-3}		0

Table S5 List of parameters and boundary conditions included in the reactive transport model. UBC= upper boundary condition, FF= fixed flux, FC= fixed concentration. All lower boundary conditions were set at no gradient.

			May-14		Jun-14		Jul-14		Sep-14	
analyte	Unit	depth (cm)	mean	s.d.	mean	s.d.	mean	s.d.	mean	s.d.
POC	m-%	0 - 5	1.6	0.6	2.3	0.3	1.1	0.7	1.9	0.5
		5 - 10	1.9	0.2	2.3	0.1	1.8	0.9	1.9	0.2
		10 - 15	1.4	0.5	1.8	0.4	1.98	0.09	1.69	0.08
		15 - 20	NA	NA	1.9	0.2	1.9	1.2	NA	NA
		20 - 25	NA	NA	1.5	0.6	NA	NA	NA	NA
PIC	m-%	0 - 5	3.3	0.65	3.6	0.25	3.5	0.7	3.2	0.7
		5 - 10	3.2	0.3	3.6	0.1	3.5	0.8	3.2	0.25
		10 - 15	3.1	0.7	3.3	0.5	3.12	0.145	3.31	0.19
		15 - 20	NA	NA	3.3	0.25	3.1	0.65	NA	NA
		20 - 25	NA	NA	3.1	0.9	NA	NA	NA	NA
PN	m-%	0 - 5	0.23	0.08	0.34	0.04	0.2	0.1	0.28	0.07
		5 - 10	0.26	0.03	0.34	0.02	0.3	0.1	0.28	0.03
		10 - 15	0.20	0.07	0.25	0.06	0.28	0.03	0.24	0.03
		15 - 20	NA	NA	0.25	0.02	0.25	0.02	NA	NA
		20 - 25	NA	NA	0.20	0.08	NA	NA	NA	NA
Fe_{tot}	mmol g-1	0 - 5	0.41	0.07	0.51	0.03	0.31	0.07	NA	NA
		5 - 10	0.43	0.04	0.55	0.02	0.35	0.08	NA	NA
		10 - 15	0.42	0.02	0.49	0.06	0.33	0.03	NA	NA
		15 - 20	NA	NA	0.42	0.04	0.32	0.02	NA	NA
		20 - 25	NA	NA	0.39	0.07	NA	NA	NA	NA
Mn_{tot}	µmol g-1	0 - 5	9	1	14	1	8	2	NA	NA
		5 - 10	10.3	0.6	13.6	0.3	10	3	NA	NA
		10 - 15	9.9	0.4	12	2	9.5	0.9	NA	NA
		15 - 20	NA	NA	10.0	0.4	9.2	0.3	NA	NA
		20 - 25	NA	NA	9	1	NA	NA	NA	NA

Table S6 average elemental composition per 5 cm for particulate organic carbon (POC), particulate inorganic carbon (PIC), particulate nitrogen (PN), total aqua regia extracteable iron (Fetot) and total aqua regia extractable manganese (Mntot). Mean is the average of two replicate cores and all measurements within that interval (see text for slicing depths), s.d. is the standard deviation of those measurements.

	average concentration in dredged sediment			**May-14**		**Jun-14**		**Jul-14**		**Sep-14**	
	1990	2000	depth (cm)	mean	s.d.	mean	s.d.	mean	s.d.	mean	s.d.
As μmol g-1	0.17	0.22	0 - 5	0.21	0.05	0.26	0.02	0.18	0.04	0.22	0.02
			5 - 10	0.23	0.02	0.274	0.007	0.20	0.04	0.29	0.03
			10 - 15	0.23	0.01	0.25	0.03	0.23	0.06	0.29	NA*
			15 - 20	NA	NA	0.22	0.02	0.18	0.03	NA	NA
			20 - 25	NA	NA	0.21	0.03	NA	NA	NA	NA
Cd μmol g-1	0.0217	0.0050	0 - 5	0.0030	0.0005	0.0031	0.0002	0.0028	0.0006	0.0023	0.0006
			5 - 10	0.0032	0.0002	0.00319	0.00008	0.0030	0.0007	0.0033	0.0003
			10 - 15	0.0035	0.0004	0.0031	0.0002	0.0033	0.0001	0.0034	0.0002
			15 - 20	NA	NA	0.0032	0.0003	0.0032	0.0007	NA	NA
			20 - 25	NA	NA	0.0032	0.0006	NA	NA	NA	NA
Cr μmol g-1	1.00	1.13	0 - 5	1.1	0.2	1.4	0.1	1.1	0.3	1.0	0.2
			5 - 10	1.1	0.1	1.54	0.07	1.2	0.4	1.2	0.1
			10 - 15	1.10	0.09	1.4	0.2	1.18	0.09	1.09	0.05
			15 - 20	NA	NA	1.1	0.1	1.0	0.1	NA	NA
			20 - 25	NA	NA	1.0	0.2	NA	NA	NA	NA
Cu μmol g-1	0.43	0.36	0 - 5	0.20	0.05	0.26	0.03	0.20	0.06	0.19	0.06
			5 - 10	0.20	0.02	0.27	0.01	0.21	0.08	0.22	0.03
			10 - 15	0.21	0.01	0.24	0.02	0.23	0.01	0.208	0.007
			15 - 20	NA	NA	0.21	0.02	0.20	0.02	NA	NA
			20 - 25	NA	NA	0.18	0.04	NA	NA	NA	NA
Pb μmol g-1	0.31	0.19	0 - 5	0.21	0.04	0.25	0.02	0.16	0.04	0.14	0.03
			5 - 10	0.22	0.03	0.27	0.02	0.17	0.05	0.19	0.02
			10 - 15	0.21	0.01	0.24	0.01	0.190	0.006	0.179	0.006
			15 - 20	NA	NA	0.22	0.03	0.16	0.02	NA	NA
			20 - 25	NA	NA	0.19	0.04	NA	NA	NA	NA
Ni μmol g-1	0.35	0.31	0 - 5	0.33	0.06	0.42	0.03	0.33	0.08	0.32	0.08
			5 - 10	0.33	0.02	0.44	0.02	0.35	0.12	0.36	0.04
			10 - 15	0.32	0.01	0.40	0.05	0.36	0.03	0.33	0.01

			15 - 20	NA	NA	0.34	0.05	0.32	0.03	NA	NA
			20 - 25	NA	NA	0.29	0.05	NA	NA	NA	NA
Zn µmol g-1	2.31	2.10	0 - 5	1.5	0. 3	1.8	0.1	1.6	0.4	1.8	0.6
			5 - 10	1.6	0.1	1.88	0.09	1.7	0.5	2.0	0.2
			10 - 15	1.61	0.06	1.7	0.1	1.9	0.1	1.95	0.05
			15 - 20	NA	NA	1.6	0.1	1.7	0.2	NA	NA
			20 - 25	NA	NA	1.5	0.3	NA	NA	NA	NA

Table S7 Overview of the average aqua regia extracteable trace metal content of the sediment cores. The trace metal content of dredged sediment from the Oostende harbour from measurements in 1990 and 2000 (http://www.mumm.ac.be/NL/Management/Sea-based/dredging.php) are given as reference. Mean is the average of two replicate cores and all measurements within that interval (see text for slicing depths), s.d. is the standard deviation of those measurements. NA values means no value could be given because the cores did not go deep enough.

***for September only 1 core was measured for As, and only 1 measurement was within the 10 – 15 cm range, thus no standard deviation could be given.**

	month	depth
POC	0.15	0.21
PIC	**‘0.03’**	0.07
PN	0.21	0.21
Fe_{tot}	0.08	0.40
Mn_{tot}	0.21	0.53
Cd	0.60	0.26
Cr	0.10	0.35
Cu	0.27	0.51
Ni	0.20	0.74
Pb	**‘0.02’**	0.35
Zn	0.34	0.38
As*	**‘0.01’**	0.35

Table S8 p-values for the two-way ANOVA, testing for month (first column) and depth zone (second column) as factors. Significant factors ($p < 0.05$) are indicated by quotation marks and in bold font.

*** for As only May, June and July where included in the ANOVA test, due to lacking duplicate in September.**

References

1. Nieuwenhuize, J., Maas, Y. E. M. & Middelburg, J. J. Rapid analysis of organic carbon and nitrogen in particulate materials. *Mar. Chem.* **45,** 217–224 (1994).
2. van de Velde, S. & Meysman, F. J. R. The influence of bioturbation on iron and sulphur cycling in marine sediments: a model analysis. *Aquat. Geochemistry* **22,** 469–504 (2016).
3. Boudreau, B. P. *Diagenetic Models and their Implementation*. (Springer-Verlag Berlin Heidelberg New York, 1997).
4. Meysman, F. J. R., Boudreau, B. P. & Middelburg, J. J. Modeling reactive transport in sediments subject to bioturbation and compaction. *Geochim. Cosmochim. Acta* **69,** 3601–3617 (2005).
5. Fick, A. Uber Diffusion. *Ann. Phys. (N. Y).* **94,** 59–86 (1855).
6. van de Velde, S., Callebaut, I., Gao, Y. & Meysman, F. J. R. Impact of electrogenic sulfur oxidation on trace metal cycling in a coastal sediment. *Chem. Geol.* **452,** 9–23 (2017).
7. van de Velde, S. *et al.* The impact of electrogenic sulfur oxidation on the biogeochemistry of coastal sediments: a field study. *Geochim. Cosmochim. Acta* **194,** 211–232 (2016).
8. Soetaert, K. & Meysman, F. Reactive transport in aquatic ecosystems: Rapid model prototyping in the open source software R. *Environ. Model. Softw.* **32,** 49–60 (2012).
9. Boudreau, B. P. A method-of-lines code for carbon and nutrient diagenesis in aquatic sediments. *Comput. Geosci.* **22,** 479–496 (1996).
10. Soetaert, K., Petzoldt, T. & Setzer, R. W. Package deSolve : Solving Initial Value Differential Equations in R. *J. Stat. Softw.* **33,** 1–25 (2010).
11. Thamdrup, B. in *Advances in Microbial Ecology* (ed. Schink, B.) 41–84 (Luwer Academic/Plenum Publishers, 2000).
12. Lohse, L., Malschaert, J. F. P., Slomp, C. P., Helder, W. & Vanraaphorst, W. Nitrogen cycling in North Sea sediments - Interaction of denitrification and nitrification in offshore and coastal areas. *Mar. Ecol. Prog. Ser.* **101,** 283–296 (1993).
13. Soetaert, K., Herman, P. M. J. & Middelburg, J. J. A model of early diagenetic processes from the shelf To abyssal depths. *Geochim. Cosmochim. Acta* **60,** 1019–1040 (1996).
14. Postma, D. & Jakobsen, R. Redox zonation: Equilibrium constraints on the

Fe(III)/SO4-reduction interface. *Geochim. Cosmochim. Acta* **60,** 3169–3175 (1996).

15. Mackin, J. E. & Aller, R. C. Ammonium adsorption in marine sediments. *Limnol. Oceanogr.* **29,** 250–257 (1984).
16. Berg, P., Rysgaard, S. & Thamdrup, B. Dynamic Modeling of Early Diagenesis and Nutrient Cycling. A Case Study in an Artic Marine Sediment. *Am. J. Sci.* **303,** 905–955 (2003).
17. Meysman, F. J. R., Middelburg, J. J., Herman, P. M. J. & Heip, C. H. R. Reactive transport in surface sediments. II. Media: an object-oriented problem-solving environment for early diagenesis. *Comput. Geosci.* **29,** 301–318 (2003).
18. Thamdrup, B., Fossing, H. & Jorgensen, B. B. Manganese , iron , and sulfur cycling in a coastal marine sediment, Aarhus Bay, Denmark. *Geochim. Cosmochim. Acta* **58,** 5115–5129 (1994).
19. Van Cappellen, P. & Wang, Y. Cycling of iron and manganese in surface sediments: a general theory for the coupled transport and reaction of carbon, oxygen, nitrogen, sulfur, iron, and manganese. *Am. J. Sci.* **296,** 197–243 (1996).
20. Fossing, H. *et al. A model set-up for an oxygen and nutrient flux model for Aarhus Bay (Denmark)*. (2004).
21. Katsev, S., Sundby, B. & Mucci, A. Modelling vertical excursions of the redox boundary in sediments: Application to deep basins of the Arctic Ocean. *Limnol. Oceanogr.* **51,** 1581–1593 (2006).
22. Meysman, F. J. R., Risgaard-Petersen, N., Malkin, S. Y. & Nielsen, L. P. The geochemical fingerprint of microbial long-distance electron transport in the seafloor. *Geochim. Cosmochim. Acta* **152,** 122–142 (2015).
23. Poulton, S. W., Krom, M. D. & Raiswell, R. A revised scheme for the reactivity of iron (oxyhydr)oxide minerals towards dissolved sulfide. *Geochim. Cosmochim. Acta* **68,** 3703–3715 (2004).
24. Rickard, D. The solubility of FeS. *Geochim. Cosmochim. Acta* **70,** 5779–5789 (2006).
25. Vasquez-Cardenas, D. *et al.* Microbial carbon metabolism associated with electrogenic sulphur oxidation in coastal sediments. *ISME J.* **9,** 1966–1978 (2015).
26. Risgaard-Petersen, N., Revil, A., Meister, P. & Nielsen, L. P. Sulfur, iron-, and calcium cycling associated with natural electric currents running through marine sediment. *Geochim. Cosmochim. Acta* **92,** 1–13 (2012).
27. Greenwood, N. N. & Earnshaw, A. *Chemistry of the Elements*. (Oxford: Pergamon Press, 1984).
